# Suppressing Mo‐Species Leaching in MoO_x_/A‐Ni_3_S_2_ Cathode for Stable Anion Exchange Membrane Water Electrolysis at Industrial‐Scale Current Density

**DOI:** 10.1002/advs.202502478

**Published:** 2025-04-30

**Authors:** Husileng Lee, Guoheng Ding, Linqin Wang, Yunxuan Ding, Tang Tang, Licheng Sun

**Affiliations:** ^1^ Center of Artificial Photosynthesis for Solar Fuels and Department of Chemistry School of Science and Research Center for Industries of the Future Westlake University 18 Shilongshan Road Hangzhou Zhejiang Province 310024 China; ^2^ Institute of Natural Sciences Westlake Institute for Advanced Study 18 Shilongshan Road Hangzhou Zhejiang Province 310024 China; ^3^ Division of Solar Energy Conversion and Catalysis at Westlake University Zhejiang Baima Lake Laboratory Hangzhou Zhejiang Province 310000 China

**Keywords:** anion exchange membrane water electrolyzer, catalyst reconstruction, hydrogen evolution reaction, large current density, MoS_2_/Ni_3_S_2_ catalysts

## Abstract

The development of non‐noble metal‐based hydrogen evolving reaction (HER) electrocatalysts operating under high current density plays a critical role in the large‐scale application of anion exchange membrane water electrolysis (AEM‐WE). Herein, a porous and hybrid MoS_2_/Ni_3_S_2_ is synthesized on nickel foam (NF) via a one‐step hydrothermal method and studied its reconstruction process during alkaline HER conditions. Experimental results indicated that the MoS_2_ underwent an oxidative dissolution followed by a dynamic equilibrium between dissolution and redeposition of the amorphous MoO_x_ during HER. Meanwhile, S‐vacancy‐rich Ni_3_S_2_ (A‐Ni_3_S_2_) is exposed and acts as the real active site for HER. The obtained MoO_x_/A‐Ni_3_S_2_ catalyst exhibited high catalytic performance in three‐electrode systems and single‐cell AEM‐WE. Finally, for a long‐term durability test in the AEM electrolyzer, a dry cathode method is applied to suppress the Mo species leaching from the MoO_x_/A‐Ni_3_S_2_ electrode. Remarkably, the device assembled by MoO_x_/A‐Ni_3_S_2_ as the cathode catalyst and NiFe as the anode catalyst demonstrated a high stability of 2500 h at 2 A cm^−2^ and 40 °C with a small aging rate of 30 µV h^−1^.

## Graphical abstract

This work develops a novel and highly efficient MoS_2_/Ni_3_S_2_ catalyst toward hydrogen evolution reaction (HER) for the anion exchange membrane water electrolyzer. During HER, the MoS_2_ on the electrode surface undergoes an oxidative dissolution and is redeposited as amorphous MoO_x_. An anion exchange membrane water electrolyzer (AEM‐WE) with MoO_x_/A‐Ni_3_S_2_ exhibits high efficiency (7.4 A cm^−2^ @ 2 V) and long durability (2500 h, 2 A cm^−2^, 40 °C).

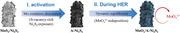

## 1 Introduction

Water electrolysis, involving the hydrogen‐evolving reaction (HER) and the oxygen‐evolving reaction (OER), has been considered one of the most sustainable approaches for producing green hydrogen (H_2_) with zero‐carbon emissions.^[^ ^1^, ^2^ ^]^ Recently, anion exchange membrane water electrolysis (AEM‐WE) has emerged as a novel technology as it can combine the merits of alkaline water electrolysis (AWE, low cost, and favorable stability) and proton exchange membrane water electrolysis (PEM‐WE, large current density and dynamic response).^[^ ^3^, ^4^ ^]^ However, the development of AEM‐WE using non‐noble catalysts is severely hindered by the low catalytic activity and stability of the anode/cathode catalysts.^[^ ^5^ ^]^ Notably, though the HER is a two‐electron process, its overpotentials (*η*) under harsh alkaline conditions have been reported to be high due to slower reaction kinetics associated with its additional energy barrier of H_2_O dissociation.^[^ ^6^, ^7^ ^]^ Additionally, under high current density (1 A cm^−2^), a large number of H_2_ bubbles generated on electrode surfaces block the active sites, further threatening the catalytic performance and limiting the efficiency of the device.^[^ ^8^, ^9^, ^10^ ^]^ Consequently, it is essential to develop highly efficient transition metal‐based HER catalysts under industrial‐relevant current density for large‐scale applications of AEM‐WE.

At present, Raney Ni serves as the predominant industrial catalyst for HER in the alkaline electrolysis system. Although it has the advantage of low cost and decent stability, its catalytic activity is still unsatisfactory, especially at high current density (500 mA cm^−2^ @ 300–500 mV).^[^ ^11^ ^]^ Thus, in past decades, numerous NiMo‐based materials have been developed as promising alternatives because of their outstanding HER performance in lab‐scale assessment. However, synthesis of the reported best HER catalysts, such as Ni_4_Mo/MoO_2_/NF,^[^ ^12^ ^]^ Pt/Ni‐Mo,^[^ ^13^ ^]^ and h‐NiMoFe,^[^ ^11^ ^]^ are still limited to the laboratory scale as they usually need a reduction step using high‐temperature annealing. As a result, such NiMo alloy catalysts are sensitive to the air and can easily be oxidized and deactivated, making it quite challenging for industrial applications.^[^ ^9^ ^]^ Besides, previous studies have uncovered the Mo dissolution in NiMo‐type catalysts (Mo in Ni_4_Mo^[^ ^14^ ^]^ and MoSe_2_^[^ ^15^ ^]^) under an alkaline HER process, which makes the deterioration matter worse.

In comparison, hybrid non‐noble metal chalcogenides, especially MoS_2_/Ni_3_S_2_, are quite stable in the air and have emerged as a potential candidate for HER catalysts.^[^ ^16^, ^17^, ^18^, ^19^, ^20^ ^]^ As widely recognized, MoS_2_/Ni_3_S_2_ is stable and synergistically catalyzes the reaction during the alkaline HER process.^[^ ^21^, ^22^ ^]^ For example, Feng et al. first reported an interface‐rich MoS_2_/Ni_3_S_2_ catalyst.^[^ ^22^ ^]^ The experimental and theoretical results displayed that the fabricated interfaces synergistically enhance the chemisorption of H* and OH^−^, facilitating the overall water splitting. Subsequently, Ren and co‐workers developed long and dense MoS_2_/Ni_3_S_2_ heterostructure nanowires on nickel foam (NF).^[^ ^23^ ^]^ Due to the porous structure and synergistic effect of MoS_2_ and Ni_3_S_2_, the catalyst exhibits excellent HER performance, affording a current density of 1 A cm^−2^ at an overpotential of 200 mV. However, due to the complicated catalytic process, especially at high current density, simply synergistic effect analysis of perfect Ni_3_S_2_ and MoS_2_ may deviate from the actual conditions, which leads to misconceived conclusions. To the best of our knowledge, the reconstruction of hybrid MoS_2_/Ni_3_S_2_ materials during HER has not been studied, and deep investigation of Mo‐species leaching is needed to improve their catalytic activity and durability.

For further practical application, new testing protocols to alleviate the Mo dissolution in AEM‐WE devices are also urgently demanded. Recently, electrode feed mode has emerged as a creative research field in the AEM‐WE.^[^ ^24^ ^]^ Because of the inherent kinetic difference between OER and HER, asymmetric feed mode could address many challenges.^[^ ^25^ ^]^ For example, Strasser's group reported an asymmetric‐feed electrolyzer design (alkaline seawater at the anode and dry cathode operation) for direct seawater electrolysis using all‐PGM‐free catalysts and cell components, avoiding the precipitation of insoluble alkaline‐earth hydroxides and carbonates on the cathode.^[^ ^26^ ^]^ As for AEM‐WE, it is expected that the dry cathode mode can not only obtain high‐purity H_2_ but also alleviate the dissolution of Mo species on the cathode. However, the anhydrous cathode mode has not been applied to the AEM‐WE devices using NiMo‐based HER catalysts.

Herein, we synthesized a hybrid MoS_2_/Ni_3_S_2_ catalyst on NF by a one‐step hydrothermal method without reduction annealing and investigated its reconstruction under alkaline HER condition. The MoS_2_ on the surface is oxidized and dissolved into the electrolyte. Then, the Mo species establish a dynamic equilibrium of dissolution and redeposition on the electrode surface. Simultaneously, Ni_3_S_2_ with abundant S‐vacancies was exposed as catalytically active sites. The resulting electrode (named MoO_x_/A‐Ni_3_S_2_) displayed high catalytic activity with an *η* of 145 mV and favorable stability over 1000 h at 1 A cm^−2^ in 1 m KOH solution. Finally, by using a dry cathode operation mode, the PGM‐free single‐cell AEM electrolyzer, assembled by the MoO_x_/A‐Ni_3_S_2_ and NiFe/NF, showed optimum durability over 2500 h at 2 A cm^−2^ at 40 °C without noticeable performance decay.

## 2 Results and Discussion

### 2.1 Synthesis and Characterization of MoS_2_/Ni_3_S_2_

**Figure 1:**
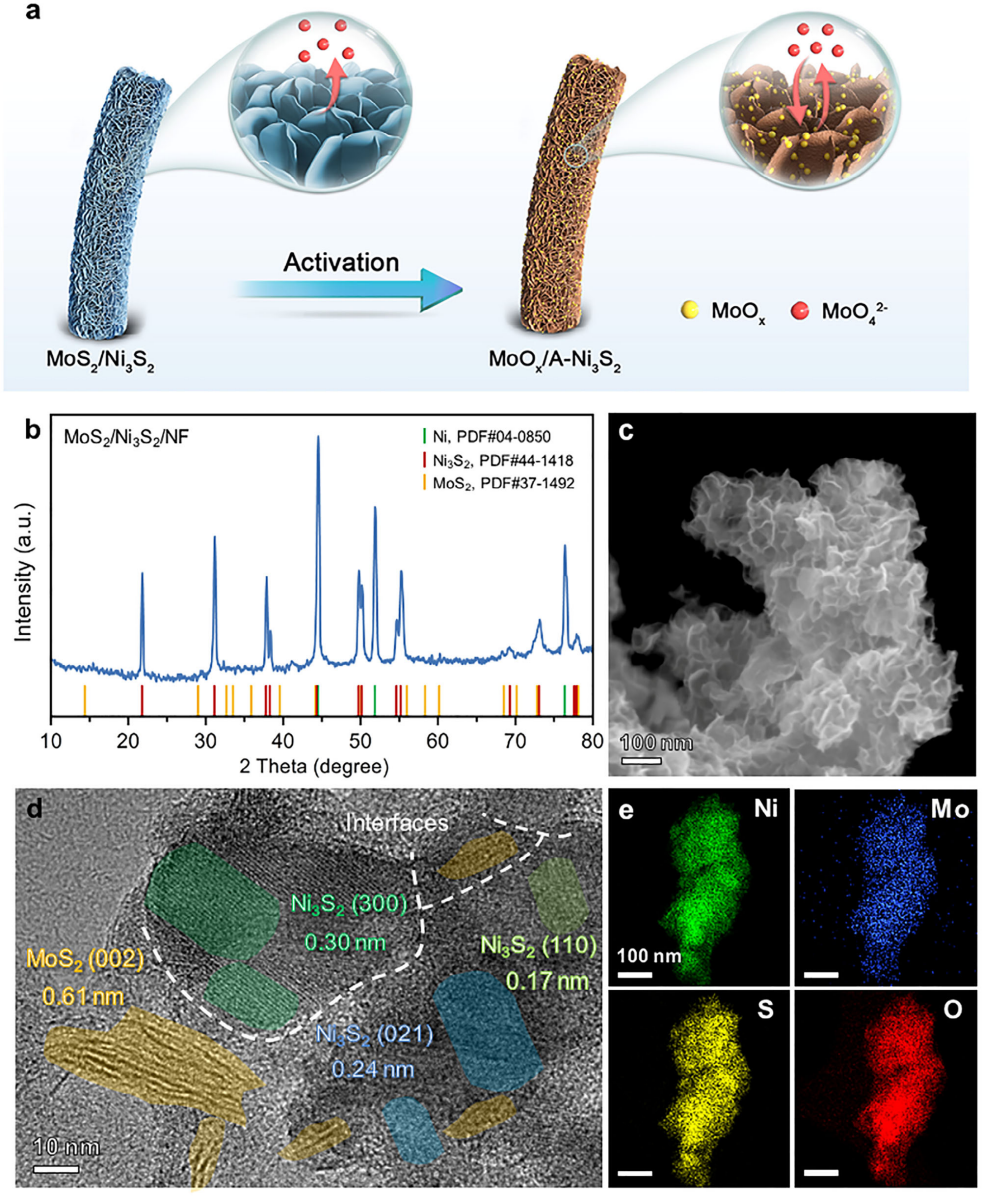
a) Schematic illustration of the fabrication of the MoO_x_/A‐Ni_3_S_2_ and its dynamic equilibrium between the dissolution and re‐absorption of MoO_x_^2−^. Characterization of as‐prepared MoS_2_/Ni_3_S_2_ on NF: b) XRD patterns, c) SEM images, d) HRTEM analysis, and e) HAADF‐STEM images and corresponding EDS elemental mapping.

The hybrid MoS_2_/Ni_3_S_2_ was typically synthesized by a one‐pot hydrothermal method. (NH_4_)_2_MoS_4_ was used as Mo and S sources, and NiCl_2_ was used as an additional Ni source. The decomposition of (NH_4_)_2_MoS_4_ generated MoS_2_, NH_3,_ and H_2_S, where H_2_S etched Ni substrate to generate Ni_3_S_2_ and MoS_2_ could easily agglomerate to form nanoflakes (Figure S1, Supporting Information). Then, the as‐obtained electrode was electrochemically activated under alkaline HER conditions. As shown in **Figure** **1**a, the MoS_2_ underwent oxidative dissolution, and S‐vacancy‐rich Ni_3_S_2_ (named A‐Ni_3_S_2_) was gradually exposed as a real active species. Finally, the MoO_x_ species was formed on the surface by a dynamic equilibrium of dissolution and redeposition process, which is beneficial for H_2_O absorption.

The as‐prepared MoS_2_/Ni_3_S_2_ electrode was characterized using various techniques. First, X‐ray diffraction (XRD) was used to detect the crystallography information of the MoS_2_/Ni_3_S_2_ catalyst (Figure 1b). Three main peaks at 44.5°, 51.9°, and 76.4° arise from the (111), (200), and (220) planes of Ni foam (Ni, PDF #04‐0850), respectively. Additionally, peaks at 21.8°, 31.1°, 37.8°, 38.3°, 49.7°, 50.1°, 54.6°, 55.2°, 69.3°, 73.0°, and 77.6° correspond to the (101), (110), (003), (021), (113), (211), (104), (122), (131), (214), and (223) planes of hexagonal Ni_3_S_2_ (PDF#44‐1418), respectively. Besides, a series of weak peaks observed at 15.5°, 29.3°, 32.7°, 33.5°, 40.2°, 59.3°, 60.5°, 69.0°, and 72.8° are attributed to the (002), (004), (100), (101), (103), (110), (008), (201), and (203) planes of 2H‐MoS_2_ (PDF#37‐1492), respectively. The morphology and components of the catalysts were further characterized using scanning electron microscopy (SEM), transmission electron microscopy (TEM), and corresponding energy dispersive X‐ray spectroscopy (EDS). As shown in SEM images (Figure 1c; Figure S2, Supporting Information), the obtained catalyst was grown on NF with porous and interconnected nanoflakes. The SEM‐EDS (Figures S3 and S4, Supporting Information) of the as‐obtained catalyst displayed that the Ni, Mo, S, and O were distributed at an approximate atomic ratio of Ni:Mo:S:O = 25:20:46:9. The cross‐section SEM images and its corresponding EDS spectrum (Figures S5 and S6, Supporting Information) showed that the homogeneous distribution of Ni, Mo, S, and O with an atomic ratio of 32:11:44:4. The high‐resolution TEM (HRTEM, Figure 1d) image of the interfacial layer showed the lattice spacing of 0.17, 0.24, 0.30, and 0.61 nm, belonging to the Ni_3_S_2_ (110), Ni_3_S_2_ (021), Ni_3_S_2_ (300), and MoS_2_ (002), respectively. In addition, the amorphous phase is inferred as MoO_x_, a usual impurity when synthesizing MoS_2_ in aqueous solution, which can also accelerate the H_2_O absorption and thus assist the HER process.^[^ ^27^ ^]^ Scanning TEM (STEM) images revealed that the size of each nanoflake is 20–50 nm, and the Ni, Mo, S, and O are uniformly distributed on the catalyst (Figure 1e). The STEM‐EDS mapping of the as‐obtained catalyst shows an atomic ratio of Ni:Mo:S:O = 22:11:24:44 (Figure S7, Supporting Information). The above results confirmed the successful fabrication of MoS_2_/Ni_3_S_2_ catalyst with interconnected nanoflakes morphology and layered structure.

### 2.2 Catalyst Reconstruction Under Alkaline HER Conditions

**Figure 2:**
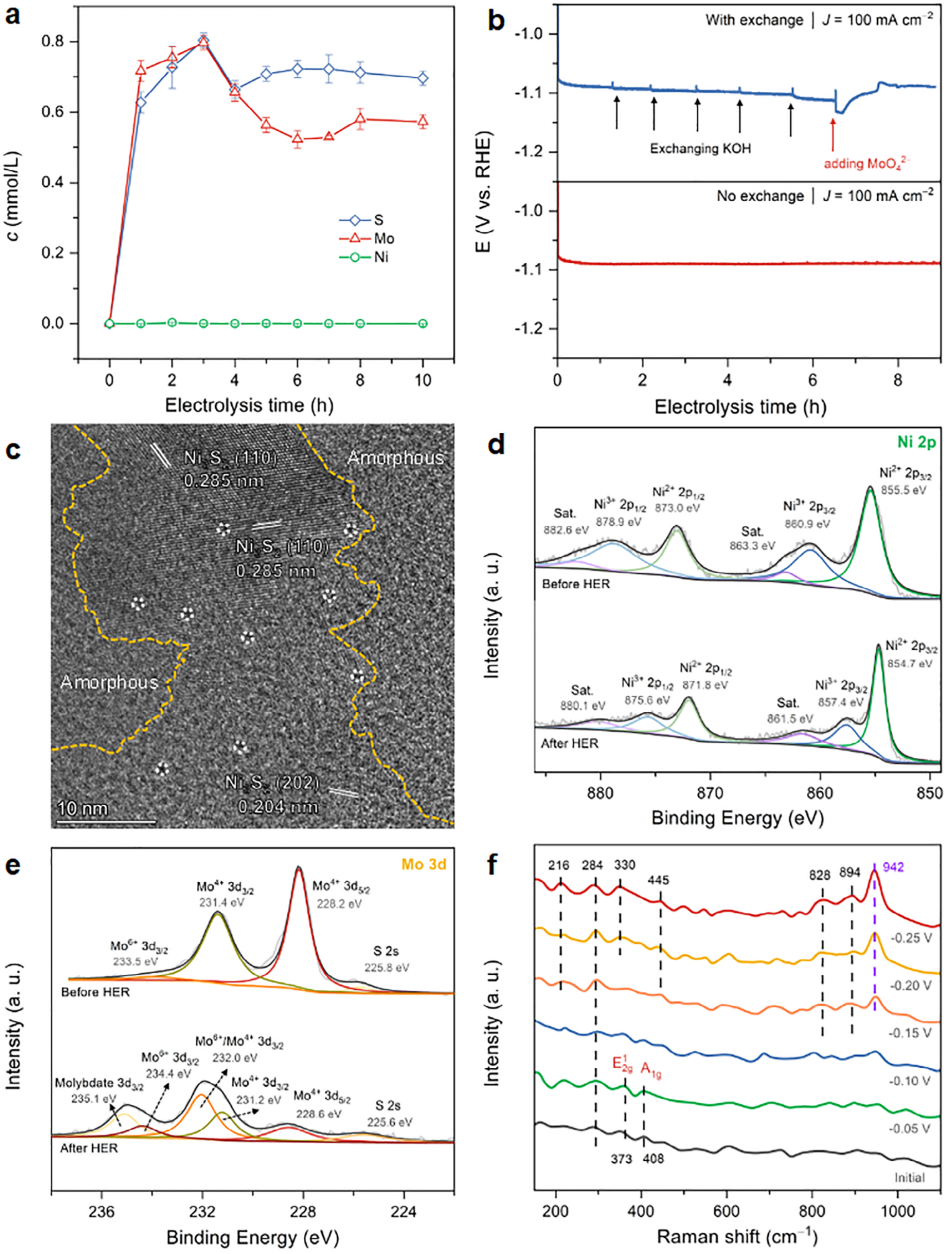
a) Time‐dependent concentration of dissolved Mo, Ni, and S in the electrolyte; b) *J‐t* curve of MoO_x_/A‐Ni_3_S_2_ with and without refreshing the electrolyte solution for every 1 h and with extra addition of Na_2_MoO_4_ in the last 2 h; c) HRTEM of MoO_x_/A‐Ni_3_S_2_; Comparison of XPS fine spectra around d) Ni 2p, e) Mo 3d before and after HER; f) In situ Raman spectra of the catalyst during HER.

The catalyst was activated by successive cyclic voltammetry (CV) scans, revealing a broad oxidation peak under reducing conditions and continuous improvement of HER activity (Figure S8, Supporting Information), which is probably caused by the electro‐oxidation of the surface species. To verify this process, we investigated the changes in the component on the electrode surface using various spectroscopic techniques during or after long‐term electrolysis. First, the Mo, Ni, and S content in the electrolyte was analyzed by inductively coupled plasma mass spectrometry (ICP‐MS) during electrolysis at 100 mA cm^−2^. As shown in **Figure** **2**a, the time‐dependent Mo and S concentration in the electrolyte sharply increases upon increasing the reaction time, and a relatively constant Ni was observed within 3 h electrolysis. After that, the content of Mo and S in the electrolyte gradually decreases and becomes steady, proving the redeposition of Mo and S on the electrode surface. After being initially activated by CV scans, the MoO_x_/A‐Ni_3_S_2_ maintains high stability at 100 mA cm^−2^ (Figure 2b). However, with continuously renewing the electrolyte solution, the stability of *J*‐*t* curves gradually deteriorated (Figure 2b), which may be caused by the break‐out of the dynamic balance of Mo species. Additionally, when 0.1 m Na_2_MoO_4_ was added into the electrolyte, the performance of the electrode improved and kept relative stability, confirming that the dynamic change of Mo species plays a vital role in the electrode's performance toward HER. Due to the dissolution and redeposition of MoO_x_ species, the electrode surface is reconstructed to a porous and hollow morphology after long‐time electrolysis (see SEM images in Figure S9, Supporting Information), which is favorable to the gas bubble release and mass transfer.

The cross‐section SEM image (Figure S10, Supporting Information) further proves that the electrode surface is changed to a porous structure while the Mo and S contents in bulk are still preserved according to the linear scan EDS, suggesting the redeposition process of MoO_x_ can prevent the further oxidation of the electrode. XRD spectrum of the post‐HER electrode (Figure S11, Supporting Information) displayed that the characteristic peaks of Ni_3_S_2_ were still preserved, though the surface morphology of the catalyst was partially broken. Furthermore, STEM images (Figure S12, Supporting Information) suggest that the nanoflake morphology is still sustained after long‐term durability tests. TEM‐EDS elemental mapping reveals that the content of Mo species was sharply decreased after HER tests, and the approximate element atomic ratio is Ni:Mo:S:O = 31:3:9:56 (Figures S12 and S13, Supporting Information), indicating the dissolution of the Mo and the reconstruction of the surface component. The HRTEM image mainly presents the lattice fringe of Ni_3_S_2_ with plenty of defects surrounded by an amorphous phase, implying the dissolution and redeposition process caused defect‐rich Ni_3_S_2_ and amorphous MoO_x_ phase (Figure 2c).

X‐ray photoelectron spectroscopy (XPS) was employed to investigate the near‐surface electron structural change of the as‐prepared material after the activation process. The XPS survey scan of pristine and activated electrodes (Figure S14, Supporting Information) suggested the existence of Ni, Mo, S, and O, which is consistent with the EDS mapping results. For the high‐resolution spectrum of Ni 2p of MoS_2_/Ni_3_S_2_ (Figure 2d), the peaks at 855.5 and 873.0 eV can be assigned to Ni 2p_3/2_ and 2p_1/2_ of Ni^2+^, while peaks at 860.9 and 878.9 eV are attributed to Ni 2p_3/2_ and 2p_1/2_ of Ni^3+^, respectively.^[^ ^28^, ^29^ ^]^ After activation, peaks ascribed to Ni^2+^ shift to lower binding energy, indicating the electron density of the Ni species slightly increases after the HER process (Figure 2d). In addition, the Mo 3d region of the as‐obtained electrode (Figure 2e) can be fitted into four peaks at 225.8, 228.2, 231.6, and 233.5 eV, which are assigned to S 2s, Mo 3d_5/2_ (Mo^4+^), Mo 3d_3/2_ (Mo^4+^), and Mo 3d_5/2_ (Mo^6+^), respectively.^[^ ^30^ ^]^ For MoO_x_/A‐Ni_3_S_2,_ the intensity of peaks related to Mo^4+^ is decreased, and a new series of peaks appear at 232.0 and 235.1 eV after CV activation, corresponding to the Mo^4+δ^ and (poly)molybdates, which confirmed that Mo re‐adsorbed on the electrode as a form of molybdates. Notably, the valence change of Ni and Mo species in activated catalyst reveals the establishment of strong electronic interaction between MoO_x_ and A‐Ni_3_S_2_, in line with the differential charge density analysis at the interface between MoO_x_ and A‐Ni_3_S_2_, which indicates a partial electron transfer from MoO_x_ to Ni_3_S_2_ (Figure S15, Supporting Information). Besides, the S 2p region (Figure S16a, Supporting Information), there are three prominent peaks located at 161.3, 162.6, and 168.1 eV, ascribed to S 2p_3/2_ and 2p_1/2_ of S^2−^ and SO_4_^2−^, respectively.^[^ ^27^, ^31^ ^]^ As expected, the peak intensity of SO_4_^2−^ increases with the decrease of the S^2−^, indicating the S leached and re‐deposited as SO_4_^2−^ during the activation process, which is in line with the ICP‐MS results. The O region of MoO_x_/A‐Ni_3_S_2_ (Figure S16b, Supporting Information) also revealed the formation of molybdates and sulfates.^[^ ^32^ ^]^ Meanwhile, to verify whether the S‐vacancies were introduced after the activation process, the samples were further characterized by Electron Paramagnetic Resonance (EPR). The prominent signal at *g* = 2.002 for MoO_x_/A‐Ni_3_S_2_ is much stronger than that of as‐prepared MoS_2_/Ni_3_S_2_ (Figure S17, Supporting Information), demonstrating that more S vacancies were introduced after electrochemical activation.

After that, the in situ Raman spectroscopy was further performed to monitor the reconstruction of Mo and Ni species during the HER process. The Raman spectrum (Figure 2f) of the as‐prepared electrode showed several peaks appear at 373 and 408 cm^−1^, which are the characteristic in‐plane E^1^_2_ _g_ and out‐of‐plane A_1_ _g_ vibrations of 2H‐MoS_2_,^[^ ^21^, ^27^ ^]^ and weak peaks at 284 cm^−1^ correspond to the A_1_ vibration of Ni‐S in Ni_3_S_2_.^[^ ^18^ ^]^ With the voltage increasing, the peak at 284 cm^−1^ shows a tendency to become sharper suggesting the exposure of Ni_3_S_2_ on the surface. Besides, as the voltage increased to −0.15 V, the characterization peaks of MoS_2_ vanished, and several new peaks appeared, suggesting that MoS_2_ on the electrode surface collapsed and reconstructed to other species. The 216, 330, and 942 cm^−1^ peaks correspond to the bending mode, diagnostic Mo−O−Mo bending mode, and terminal Mo = O bond of polymolybdate species, Mo_2_O_7_^2−^.^[^ ^33^, ^34^ ^]^ Peaks at 828 and 894 cm^−1^ are indexed to the antisymmetric stretching mode of Mo−O−Mo and Mo = O of MoO_x_. In addition, a peak at 445 cm^−1^ is associated with the *ν*_2_ symmetric bending of SO_4_^2−^, indicating that the S in MoS_2_ is transferred to sulfates.^[^ ^35^ ^]^ The Pourbaix diagram of MoS_2_ also reveals that the MoS_2_ exists as MoO_4_^2−^ + S^2−^ state under HER conditions at pH 14.^[^ ^36^ ^]^ Based on the above investigation, we can conclude that MoS_2_ is first oxidatively dissolved into the electrolyte as MoO_4_^2−^, which is redeposited on the electrode surface as MoO_x_ under negative potential (The proposed mechanism see Supporting Information). After that, the MoO_x_ establishes a dynamic equilibrium between dissolution and redeposition.^[^ ^14^, ^37^ ^]^ Meanwhile, the A‐Ni_3_S_2_ is exposed, which is expected as the real catalytically active site.

### 2.3 Half‐Cell HER Performance of the MoO_x_/A‐Ni_3_S_2_

**Figure 3:**
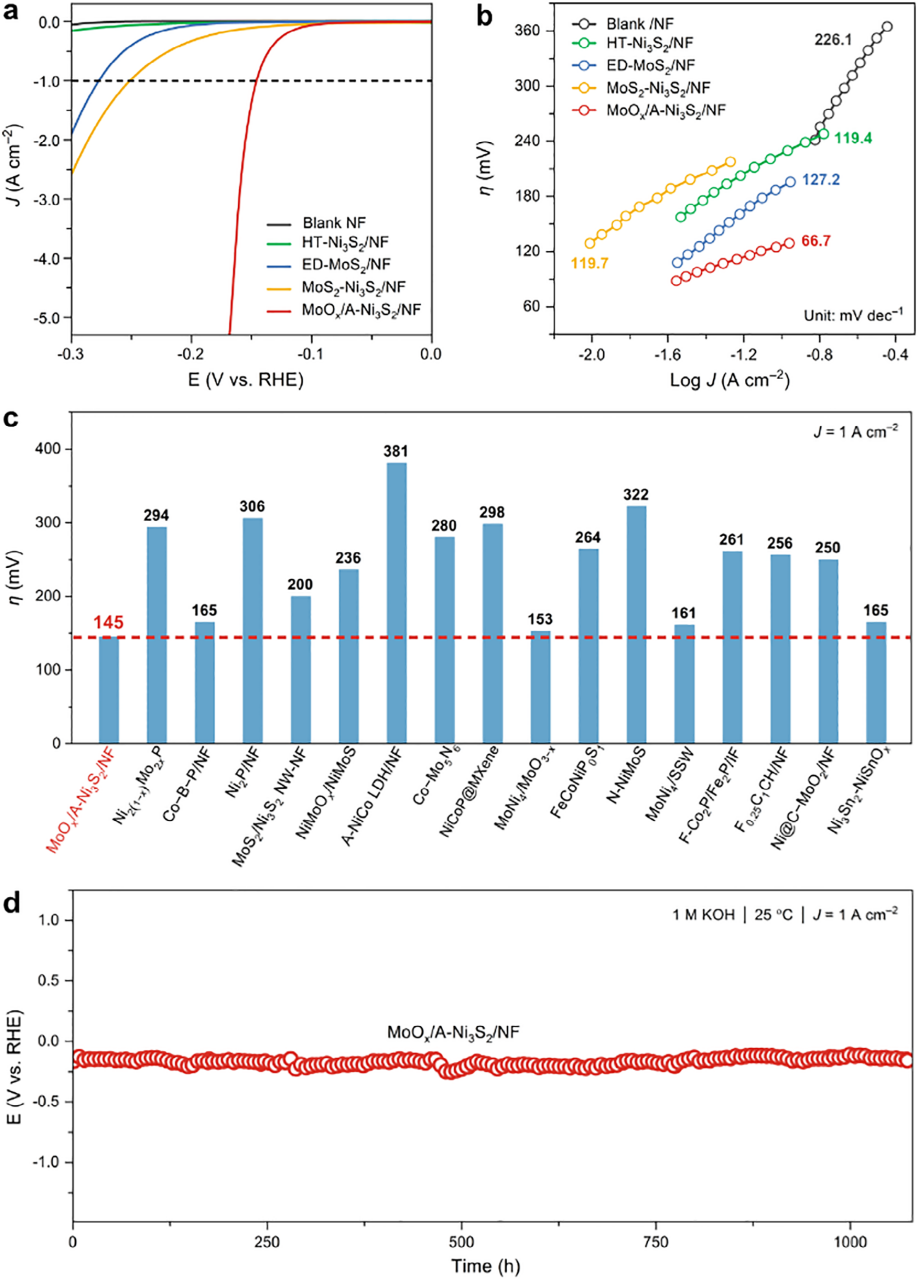
a) Polarization curves (with 90% *iR*‐compensation) and b) Tafel slopes of the different electrodes in 1 m KOH; c) Comparison of the overpotential (*η*) at 1 A cm^−2^ between MoO_x_/A‐Ni_3_S_2_ and reported HER catalysts; d) Chronopotentiometric curve of the MoO_x_/A‐Ni_3_S_2_ at 1 A cm^−2^ in 1 m KOH solution.

Then, the electrocatalytic HER measurements of the electrodes were evaluated using a typical three‐electrode system in a 1 m KOH electrolyte at 25 °C. First, the hydrothermal temperature/time, reactant ratio, and DMF amount were carefully optimized (Figure S18, Supporting Information). Besides, for comparison, other samples were also prepared parallelly (see methods in the Supporting Information): 40% Pt/C/NF, hydrothermally synthesized Ni_3_S_2_ nanoplates (HT‐Ni_3_S_2_/NF), electrodeposited MoS_2_ nanoparticles (ED‐MoS_2_/NF), and electrodeposited MoS_2_ on hydrothermal Ni_3_S_2_ (MoS_2_‐Ni_3_S_2_/NF). As depicted in the polarization curves (**Figure** **3**a), the activated catalyst exhibited the lowest overpotential (*η*) of 145 mV to deliver a current density of 1 A cm^−2^, surpassing those of HT‐Ni_3_S_2_/NF (397 mV), ED‐MoS_2_/NF (277 mV), MoS_2_‐Ni_3_S_2_/NF (250 mV), and blank NF (402 mV). Although MoO_x_/A‐Ni_3_S_2_ shows inferior catalytic performance to 40% Pt/C/NF at lower current density, it requires lower *η* when the current density is higher than 0.5 A cm^−2^, suggesting that the catalyst showed faster charge and mass transfer under high current density (Figure S19, Supporting Information). Furthermore, the Tafel plots (Figure 3b) were obtained from the polarization curves to study the fundamental mechanism. The Tafel slope of MoO_x_/A‐Ni_3_S_2_ is 66.7 mV dec^−1^, indicating it follows the Volmer–Heyrovsky mechanism, lower than those of HT‐Ni_3_S_2_ /NF (119.4 mV dec^−1^), ED‐MoS_2_/NF (127.2 mV dec^−1^), MoS_2_‐Ni_3_S_2_/NF (119.7 mV dec^−1^), and blank NF (226.1 mV dec^−1^). The above results confirmed that the unique MoO_x_/A‐Ni_3_S_2_ catalyst exhibits superior catalytic performance compared to Ni_3_S_2_, MoS_2_, and their simple combination. Moreover, the catalytic activity of the MoO_x_/A‐Ni_3_S_2_ catalyst is superior to most of the reported HER catalysts (Figure 3c and Table S1, Supporting Information). The Faradic efficiency of MoO_x_/A‐Ni_3_S_2_ reached ≈100% by comparing the amount of H_2_ during the electrolysis at 100 mA cm^−2^ with the theoretical value (Figure S20, Supporting Information). Additionally, the chronopotentiometric result (Figure 3d) presented that the as‐fabricated electrode can maintain activity for at least 1200 h when electrolyzed at the constant current density of 1 A cm^−2^.

To illustrate the role of the redeposited MoO_x_ species and the reason contributing to the excellent catalytic performance of MoO_x_/A‐Ni_3_S_2_, the double‐layer capacitance values (*C*_dl_, Figures S21 and S22, Supporting Information) of all samples were first measured using the cyclic voltammetry (CV) technique to determine the electrochemical surface area (ECSA). MoO_x_/A‐Ni_3_S_2_ has a considerably higher *C*_dl_ (30.5 mF cm^−2^) than those of 40% Pt/C/NF (23.7 mF cm^−2^), HT‐Ni_3_S_2_/NF (0.54 mF cm^−2^), ED‐MoS_2_/NF (3.14 mF cm^−2^), MoS_2_‐Ni_3_S_2_/NF (1.39 mF cm^−2^), and blank NF (0.21 mF cm^−2^), which suggests that the catalyst exposes more electrochemical active sites for HER. After that, the electrochemical impedance spectroscopy (EIS) was obtained and fitted using an equivalent circuit to investigate the charge and mass transfer characteristics of all electrodes at a bias of −0.16 V. Based on the Nyquist plots and the fitting results (Figure S23 and Table S2, Supporting Information), the charge transfer resistance (*R*_ct_) and mass transfer resistance (*R*_mt_) of MoO_x_/A‐Ni_3_S_2_ were much lower than those of control samples, indicating the in situ generation and unique reconstruction process of the MoO_x_/A‐Ni_3_S_2_ are in favor of fast charge and mass transfer during HER. Besides, the gas bubble adhesive force measurements were further carried out to supply direct evidence for illustrating the bubble detachment behavior of the catalysts. As shown in Figure S24 (Supporting Information), the MoO_x_/A‐Ni_3_S_2_ has a lower adhesive force (9.1 µN) than that of as‐prepared MoS_2_/Ni_3_S_2_ (21.8 µN), which indicates the re‐deposited MoO_x_ is beneficial for the gas bubble release. Additionally, the diameter distribution of bubbles on the as‐prepared MoS_2_/Ni_3_S_2_ and MoO_x_/A‐Ni_3_S_2_ was tested at a current density of 50 mA cm^−2^ using a frame‐to‐frame camera. The H_2_ bubbles on MoO_x_/A‐Ni_3_S_2_ distributed more evenly and had smaller average bubble diameters than those of as‐prepared MoS_2_/Ni_3_S_2_ (Figure S25, Supporting Information). Furthermore, different molar Na_2_MoO_4_ was introduced to the electrolyte when evaluating the catalytic performance of HT‐Ni_3_S_2_, and the results indicated that a proper amount of MoO_4_^2−^ can also improve the catalytic activity and stability of HT‐Ni_3_S_2_ (see Figures S26 and S27and Notes, Supporting Information). To sum up, such a dynamic equilibrium between the dissolution and redeposition process of Mo species not only exposes the catalytically active S‐vacancy‐rich Ni_3_S_2_ phase during the HER process but also benefits the durability of the activated catalyst.

Finally, first‐principles density functional theory (DFT) simulations were performed to obtain an in‐depth understanding of the structure‐activity relationships of the MoO_x_/A‐Ni_3_S_2_ (Figure S28, Supporting Information). To verify whether Ni_3_S_2_ is the real active species, the reaction‐free energies of alkaline HER steps on pristine Ni_3_S_2_ and A‐Ni_3_S_2_ (110) surfaces were first calculated (Figure S29, Supporting Information). Obviously, the rate‐determining step (RDS) of Ni_3_S_2_ is the Volmer step (H_2_O + e^−^ → H* + OH^−^) with an reaction‐free energy of 1.81 eV, which is in agreement with the experimentally measured Tafel slope of 119.4 mV dec^−1^. In comparison, the RDS of A‐Ni_3_S_2_ is the Heyrovsky step (H_2_O + H* + e^−^ → H_2_ + OH^−^), in line with the Tafel slope of 66.7 mV dec^−1^. Notably, the reaction‐free energy of RDS on A‐Ni_3_S_2_ is largely reduced to 0.55 eV. Moreover, the adsorption‐free energies of H_2_O (ΔE_water_) on the MoO_x_, Ni_3_S_2_, A‐Ni_3_S_2_, and MoO_x_/A‐Ni_3_S_2_ surfaces were calculated to identify the H_2_O adsorption configurations (Figures S30 and S31, Supporting Information). The result shows that H_2_O is more prone to be adsorbed on the Mo site of MoO_x_/A‐Ni_3_S_2_ with the ΔE_water_ of −0.66 eV than on Ni_3_S_2_ (ΔE_water_ = 0.32 eV), MoO_x_ (ΔE_water_ = −0.04 eV), and A‐Ni_3_S_2_ (ΔE_water_ = −0.16 eV). This indicates that the water concentration can be enriched on the heterojunction between MoO_x_/A‐Ni_3_S_2_, in which the water is easily transferred to the A‐Ni_3_S_2_. To sum up, redeposited MoO_x_ species on the surface serve as an effective mediator for H_2_O adsorption and transfer, while the S‐vacancy‐abundant A‐Ni_3_S_2_ acts as the actual active site for HER.

### 2.4 Single‐Cell AEM‐WE Tests Using MoO_x_/A‐Ni_3_S_2_

**Figure 4:**
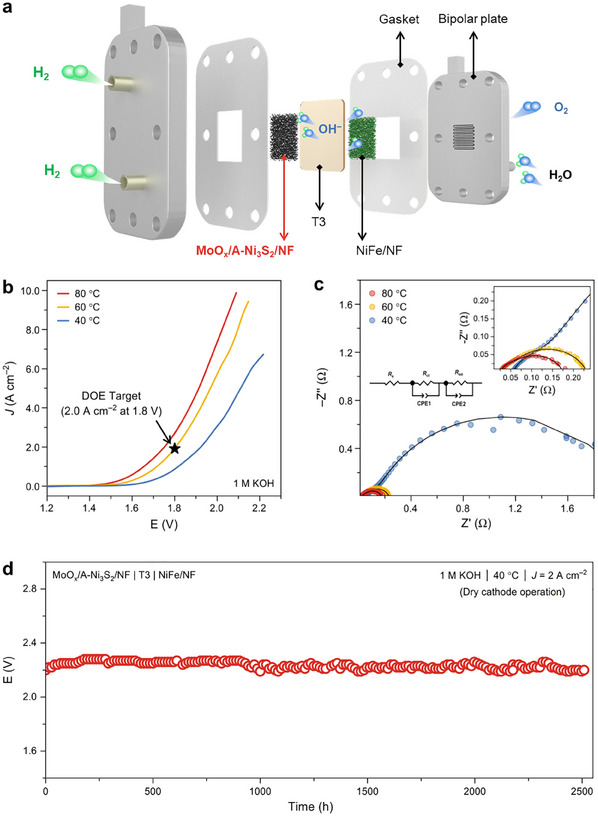
a) The schematic illustration of single‐cell AEM electrolyzer; b) The polarization curves and c) EIS spectra of “NiMo||NiFe” at different temperatures; inset: equivalent circuit models used to fit EIS; d) The chronopotentiometric curves of single‐cell AEM‐WE at a current density of 2 A cm^−2^ at 40 °C; T3, NiFe, and MoO_x_/A‐Ni_3_S_2_ were used as AEM, anode, and cathode, respectively.

Encouraged by the excellent activity and stability in half‐cell tests, we further coupled the MoO_x_/A‐Ni_3_S_2_ with a non‐noble NiFe‐based OER catalyst to construct single‐cell AEM‐WE (**Figure** **4**a). In the single cell, MoO_x_/A‐Ni_3_S_2_, NiFe/NF,^[^ ^38^ ^]^ and T3^[^ ^39^ ^]^ were used as cathode, anode, and membrane, respectively, which is called “NiMo||NiFe”. Upon conditioning at 500 mA cm^−2^ for 30 min, the polarization curves were obtained at different temperatures with no *iR* compensation (Figure 4b). The performance of both devices improved as the temperature increased. The “NiMo||NiFe” delivered current densities of 2.06 A cm^−2^ (60 °C) and 2.74 A cm^−2^ (80 °C) at the cell voltage of 1.8 V, exceeding the target of the alkaline water electrolyzer (2.0 A cm^−2^ at 1.8 V) proposed by the US Department of the Energy (DOE).^[^ ^39^ ^]^ Notably, the single‐cell can reach a current density of 7.4 A cm^−2^ at the cell voltage of 2.0 V at 80 °C, outstripping most of the reported AEM‐WEs assembled with non‐noble metal‐based catalysts (Table S3, Supporting Information). Additionally, EIS was carried out at an applied cell voltage of 1.6 V to investigate the cell resistance. Nyquist plots were plotted and fitted using an equivalent electrical circuit. As displayed in Figure 4c, the ohmic resistance (*R*_s_) of “NiMo||NiFe” is influenced slightly by the temperature. The charge transfer resistance (*R*_ct_) and mass transfer resistance (*R*_mt_) significantly decrease as the temperature increases due to the fast kinetic of the catalysts.

Long‐term durability is another critical factor when evaluating the performance of AEM‐WE. The “NiMo||NiFe” can sustain a current density of 1 A cm^−2^ at room temperature for 220 h with a voltage increase rate of 695 µV h^−1^ (Figure S32, Supporting Information). The performance decline rate from 0 to 120 h (1.18 mV h^−1^) is much faster than that from 100 to 220 h (0.1 mV h^−1^), which could be due to the more rapid dissolution of Mo, leading to the faster deterioration of HER activity in the early stage. To alleviate the dissolution of Mo and guarantee its local concentration, the alkaline electrolyte was exclusively fed at the anode (dry cathode mode). For the dry cathode operation, H_2_O was transferred from the anode to the cathode through the membrane, HER occurred, and then the generated OH^−^ was returned to the anode. Consequently, the reconstructed cathode would not be continuously flushed by the catholyte, which is beneficial for the durability of our catalyst. Besides, considering the cathode and membrane interface have a neutral or weak alkaline environment, we initially conducted durability tests of MoO_x_/A‐Ni_3_S_2_ in 1.0 M neutral (pH 7.0) and weak alkaline (pH 9.0) phosphorous buffer solution (PBS), and the results showed that the electrode also performs favorable stability at 1 A cm^−2^ under both test conditions (Figures S33 and S34, Supporting Information). Based on the above results, the durability test of “NiMo||NiFe” was further performed at a current density of 2 A cm^−2^ at 40 °C using dry cathode operation. In this circumstance, the cell can operate for 2500 h with only a 30 µV h^−1^ voltage increase rate (Figure 4d). Besides, the CV curves and Nyquist plots of “NiMo||NiFe” indicated that the performance of the MEA has only slightly decreased after 2000 h of electrolysis (Figure S35, Supporting Information). Furthermore, ICP‐MS was conducted to monitor the content of Ni, Fe, and Mo in the anolyte during 100 h electrolysis at a current density of 1 A cm^−2^ at room temperature using dry cathode operation. As can be seen in Figure S36 (Supporting Information), the time‐dependent Mo concentration in the electrolyte increases upon increasing the reaction time, indicating the MoO_4_^2−^ is transferred through the membrane to the anolyte. However, the Mo content is much less than the Mo content in the electrolyte of the half‐cell electrolyzer (Figure 2a), even though the electrolysis was conducted for over 100 h. Overall, such a dry cathode mode alleviated the continuous dissolution of Mo species and efficiently improved the stability of the single cell.

## 3 Conclusion

In summary, a porous and heterostructured MoS_2_/Ni_3_S_2_ was synthesized on the surface of NF via a one‐step hydrothermal method, which established a dynamic equilibrium of dissolution/redeposition on the electrode surface while catalytically active Ni_3_S_2_ was gradually exposed. The resulting MoO_x_/A‐Ni_3_S_2_ exhibited high efficiency and durability toward HER in harsh alkaline solution, affording current densities of 1 A cm^−2^ at an overpotential of 145 mV and maintaining a current density of 1 A cm^−2^ at least for 1200 h. Remarkably, the single‐cell AEM electrolyzer with MoO_x_/A‐Ni_3_S_2_ cathode and NiFe‐based anode exhibited a high current density of 7.4 A cm^−2^ at 2 V_cell_ at 80 °C and favorable stability at 2 A cm^−2^ at 40 °C for at least 2500 h using dry cathode mode. This study provides new insight into the reconstruction mechanism for MoS_2_/Ni_3_S_2_ materials during the alkaline HER process and proposes a practical protocol for alleviating the dissolution of Mo species.

## Conflict of Interest

The authors declare no conflict of interest.

## Supporting information



Supporting Information

## Data Availability

The data that support the findings of this study are available in the supplementary material of this article.
