# Supplementary material for: Suppressing Mo‐Species Leaching in MoOx/A‐Ni3S2 Cathode for Stable Anion Exchange Membrane Water Electrolysis at Industrial‐Scale Current Density
Source: Adv Sci (Weinh). 2025 Apr 30;12(27):2502478. doi: 10.1002/advs.202502478 (PMC12279178; doi:10.1002/advs.202502478)
Supplement: Supplementary file 1 — Supporting Information [file ADVS-12-2502478-s001.docx]

Supporting Information

**Suppressing Mo-species Leaching in MoO_x_/A-Ni_3_S_2_ Cathode for Stable Anion Exchange Membrane Water Electrolysis at Industrial-Scale Current Density**

Husileng Lee^a,b,+^, Guoheng Ding^a,b, +^, Linqin Wang^a,b^, Yunxuan Ding^a,b^, Tang Tang^a,b^, Licheng Sun^a,b,c,*^

^a^ Center of Artificial Photosynthesis for Solar Fuels and Department of Chemistry, School of Science, and Research Center for Industries of the Future, Westlake University,18 Shilongshan Road, Hangzhou 310024, Zhejiang Province, China.

^b^ Institute of Natural Sciences, Westlake Institute for Advanced Study, 18 Shilongshan Road, Hangzhou 310024, Zhejiang Province, China.

^c^ Division of Solar Energy Conversion and Catalysis at Westlake University, Zhejiang Baima Lake Laboratory, Hangzhou 310000, Zhejiang Province, China.

E-mail: sunlicheng@westlake.edu.cn.

[+] These authors contributed equally to this work.

**Materials and Instrumentals**

Ni foam (NF, thickness: 1 mm, mass density: 1500 g m^‒2^, 100 ppi) was purchased from Suzhou Xingzhenghong Metal foam Co., Ltd. *N,N*-Dimethylformamide (DMF, CAS: 68-12-2), Nickel (II) chloride hexahydrate (NiCl_2_·6H_2_O, CAS: 7791-20-0) and ammonium tetrathiomolybdate ((NH_4_)_2_MoS_4_, CAS: 15060-55-6) were purchased from J&K Scientific Co., Ltd. Potassium hydroxide (KOH, CAS: 1310-58-3) were purchased from Shanghai Macklin Biochemical Co. Ltd. 40% Pt/C were purchased from Suzhou Sinero Technology Co., Ltd. Thiourea (CS(NH_2_)_2_, CAS: 62-56-6) was purchased from Sigma Aldrich Chemie Gmbh. Nickel (II) nitrate hexahydrate (Ni(NO_3_)_2_·6H_2_O, CAS: 13478-00-7), sodium molybdate dihydrate (Na_2_MoO_4_·2H_2_O, CAS: 10102-40-6), ferrous sulfate heptahydrate (FeSO_4_·7H_2_O, CAS: 7782-63-0), and isopropanol (C_3_H_8_O, CAS: 67-63-0) were purchased from Shanghai Titan Scientific Co., Ltd. Highly purified water (> 18 MΩ·cm resistivity) was obtained from a Plus-E2-30TJ system and used throughout the experiments.

The powder X-ray diffraction (PXRD) patterns were recorded on a Bruker D8 Advance diffractometer with Cu K*α* radiation. Scanning electron microscopy (SEM) images and their corresponding energy dispersive X-ray spectroscopy (EDX) were obtained using a Zeiss 450 field emission scanning electron microscope. The cross-section of the catalysts was prepared and observed via a focused ion beam scanning electron microscope (FIB-SEM, Helios 5 UX, Thermo Fisher Scientific) with Ga^+^ as the ion source. To protect the catalysts from being damaged by the electron/ion beams, a carbon layer of ~2 μm was deposited before cutting and milling. The images of the transmission electron microscope (TEM), high-resolution transmission electron microscope (HRTEM), selected area electron diffraction (SAED) and EDX spectrum were obtained with the Talos F200X G2 (Thermo Fisher Scientific) field emission high-resolution TEM at an operating voltage of 200 kV. The X-ray photoelectron spectroscopy (XPS) was carried out on a Thermo Scientific/ESCALAB Xi+ X-ray photoelectron spectrometer. Raman spectroscopy was recorded on the mentioned Renishaw via reflex Raman microscope under an excitation of 532 nm laser at controlled potentials by an electrochemical workstation. The electrolytic cell was a home-made device, where the working electrode (the freshly prepared Ni_3_S_2_/MoS_2_) was kept perpendicular to the incident laser, Pt wire as the counter electrode was rolled to a circle around the working electrode, and the Hg/HgO electrode with an inner reference electrolyte of 1 M KOH was used as the reference electrode. The applied potential should be carefully controlled to avoid generating too many bubbles to block the light path. The inductively coupled plasma mass spectrometry (ICP-MS) was carried out on a Thermo Fisher iCAP RQ mass spectrometer. The 0.5 mL of the electrolyte was taken out for the measurement, and 0.5 mL of 1 M KOH was added immediately to maintain the ionic concentration. A frame-to-frame camera was used to record the bubble behavior over the as-prepared MoS_2_/Ni_3_S_2_ and MoO_x_/A-Ni_3_S_2_, where a three-electrode system (working electrode: MoS_2_/Ni_3_S_2_ or MoO_x_/A-Ni_3_S_2_, counter electrode: Pt mesh, reference electrode: Hg/HgO) was applied for observation in 1 M KOH at a current density of 50 mA cm^‒^². The interaction force between gas bubbles and electrode (MoS_2_/Ni_3_S_2_ or MoO_x_/A-Ni_3_S_2_) was measured by a high sensitivity micro-electro-mechanical balance system (Dataphysics DCAT25, Germany). A high-speed camera captured the measurement process. The gas bubble was suspended on a metal needle in 1 M KOH. Subsequently, the electrode moved upward and contacted the gas bubble. When the overlaid substrate left the gas bubble, the force gradually increased and reached a critical point. Finally, the overlaid substrate detached from the bubble, and the adhesive force between the electrodes and the gas bubble was measured.

**Preparation of MoS_2_/Ni_3_S_2_**

The MoS_2_/Ni_3_S_2_ was prepared using a one-step hydrothermal method. Before synthesis, NF was cleaned ultrasonically with 3 M HCl, DI water and ethanol for 30 min each. For preparing the solution, 5 mL solution of 6 mM NiCl_2_·6H_2_O was added dropwise to 20 mL aqueous solution of 20 mM (NH_4_)_2_MoS_4_ and stirred for 30 min with bubbling N_2_. In a typical procedure, the cleaned NF was put into a 50 mL Teflon-lined stainless autoclave containing 35 mL of solution. The autoclave was sealed and maintained at 180 °C for 8 h. The resulting material was washed with ethanol three times and dried in vacuum at room temperature. Meanwhile, some critical parameters, such as reaction temperature, reaction time, water/DMF ratio, and Ni/Mo ratio, were optimized using similar methods. The optimized mass loading of the electrocatalyst is about 10.5 mg cm^−2^.

**Preparation of MoO_x_/A-Ni_3_S_2_**

The freshly prepared MoS_2_/Ni_3_S_2_ was further activated by 20 successive cyclic voltammetry (CV) scans in a potential range of 0 ~ ‒0.6 V *vs.* RHE at a scan rate of 50 mV s^−1^. In the system, MoS_2_/Ni_3_S_2_, NF, and Hg/HgO were used as working, counter, and reference electrodes, respectively.

**Preparation of ED-MoS_2_/NF**

According to the literature report, the MoS_2_ was electrodeposited on NF^[1]^. Before electrodeposition, NF was cleaned ultrasonically in 3 M HCl, ethanol and acetone. The electrodeposition was conducted in the 70 mL electrolyte containing 10 mM (NH_4_)_2_MoS_4_ and 0.2 M KCl, using NF, Pt foil and Ag/AgCl as working, counter, and reference electrode, respectively. The electrodeposition was performed at constant potential at ‒1.1 V *vs.* E_Ag/AgCl_ for 300 s. Finally, the as-obtained electrodes were alternately rinsed with deionized water and ethanol several times and dried in vacuum before use.

**Preparation of HT-Ni_3_S_2_/NF**

Ni_3_S_2_/NF was synthesized according to previously reported procedures^[2]^. Before the preparation, NF was cleaned ultrasonically in 3 M HCl, ethanol and acetone. A piece of NF (1 × 3 cm^2^) was submerged into a 25 mL Teflon-lined stainless autoclave containing 15 mL of 6.25 mmol Na_2_S ethanol solution. The autoclave was sealed and maintained at 120 °C for 20 h. The resulting material was washed with ethanol and dried in vacuum at room temperature, leading to Ni_3_S_2_/NF.

**Preparation of MoS_2_-Ni_3_S_2_/NF**

MoS_2_-Ni_3_S_2_/NF was prepared using a two-step method. The Ni_3_S_2_ was first prepared on NF using the hydrothermal method, and then MoS_2_ was electrodeposited on it according to the abovementioned procedure.

**Preparation of Pt/C/NF**

40% Pt/C powder and i-T1 (20 *wt*% of catalyst) were dispersed homogenously in 1 mL mixture of isopropyl alcohol and ultrapure water (v/v = 1/1) by sonication. Then, the defined volume ink was sprayed by a spray gun on the surface of 1 cm^2^ NF and dried in vacuum at room temperature (loading: 0.5 mg cm^−2^).

**Preparation of NiFe catalyst**

The catalyst was prepared using our previously reported procedures with modifications ^[3]^. Before the preparation, NF was cleaned ultrasonically in 3 M HCl, ethanol and acetone. Firstly, a 3D porous Ni layer was electrodeposited on the surface of the cleaned NF. The electrodeposition was conducted in NiCl_2_·6H_2_O (0.1 M) and NH_4_Cl (2 M) solution using a two-electrode cell using the NF (4 cm^2^) as anode and Pt mesh (4 cm^2^) as cathode, respectively. The 3D porous Ni layer was deposited at a cathodic current density of ~ 1.5 A cm^−2^ for 80 s, labeled Ni/NF. Then, the NiFe catalyst was deposited using a soaking method. Briefly, 1.43 g Ni(NO_3_)_2_·6H_2_O and 0.195 g FeSO_4_·7H_2_O were dissolved in 24 mL *i*-PrOH and 8 mL deionized water, respectively. The solutions above were mixed under vigorous stirring to form the heterogeneous nucleation (HN) liquid. After sonicating, the Ni/NF was then immersed into the HN liquid at 25 °C for 24 h, followed by washing with deionized water and drying in a vacuum oven at 25 °C for 12 h to generate the NiFe catalyst.

**Electrochemical Measurements**

(1) Three-electrode System

The electrochemical measurements in three-electrode systems were performed using Autolab PGSTAT302N. The catalyst was directly used as the working electrode with a geometric area of 1 cm^2^ for HER tests. A Hg/HgO electrode in 1 M KOH solution and a Pt electrode were used as reference and counter electrodes, respectively. The Hg/HgO electrode was calibrated by testing the cyclic voltammetry (CV) of the (Ru(bpy)_3_)^3+^/(Ru(bpy)_3_)^2+^ aqueous solution on a glass carbon electrode as a working electrode. The calculated equation is **Equation 1** and E_Hg/HgO_ = 0.091 V *vs.* SHE. All the potential versus reversible hydrogen electrodes of the catalysts were calculated according to **Equation 2**. The pH of prepared 1 M KOH solutions varied from 13.56 to 13.89.

$E_{HgO/Hg}= E_{({{\mathrm{Ru}\left( \mathrm{bpy} \right)}_{3})}^{3+}/({{\mathrm{Ru}\left( \mathrm{bpy} \right)}_{3})}^{2+}}^{ө}-E_{\mathrm{measured}}$ (1)

$E_{versus RHE}= E_{\mathrm{measured}}+ E_{Hg/HgO} +0.059 pH$ (2)

The set potential range for linear sweep voltammetry (LSV) measurements is from ‒1.4 to ‒0.9 V (versus E_Hg/HgO_) with a scan rate of 5 mV s^‒1^. The Tafel plots were obtained by a variety of bulk electrolysis in 1 M KOH, and the stable currents were recorded at the voltages achieving 10 ~ 100 mV in every 10-mV step for 300 s constant potential electrolysis experiments with gentle stirring. The *R*_s_ values were extracted from electrochemical impedance spectroscopy (EIS), which was performed at the potential where the current density of catalysts arrived at ‒1.06 V (versus E_Hg/HgO_) with an amplitude of 5 mV and scanning frequency ranging from 100 kHz to 0.01 Hz. The electrochemical double layer capacitance was performed by CV at potentials ranging from ‒0.35 to ‒0.25 V (versus E_Hg/HgO_) with various scan rates of 20, 40, 60, 80, 100 mV s^‒1^. By plotting the difference in current density (Δ*j*/2) between the anodic and cathodic sweeps at ‒0.3 V (versus E_Hg/HgO_) against the scan rate, a linear trend was observed. The slope of the fitting line is found to be equal to twice the geometric double-layer capacitance (*C*_dl_). A water drainage method was used to measure the Faradic efficiency. The actual moles of hydrogen (H_2_) generated were calculated from the volume of collected H_2_. The theoretical moles were calculated according to the Faraday law.

(2) Single-cell AEM-WE Tests

The electrochemical measurements in AEM-WE devices were performed using an Autolab PGSTAT302N. The NiFe catalyst and MoO_x_/A-Ni_3_S_2_ were used as anodes and cathodes with a working area of 1 cm^2^, respectively. The type of anion exchange membrane used is T3. The performance of the AEM devices was recorded in 1 M KOH at 40, 60 and 80 °C. For LSV measurements, the set potential range is from 2.4 to 1.0 V with a scan rate of 5 mV s^‒1^. The EIS was performed at 1.60 V with an amplitude of 5 mV and scanning frequency ranging from 100 kHz to 0.1 Hz. Long-term durability was tested at “room temperature, 1 A cm^‒2^” and “40 °C, 2 A cm^‒2^”. The electrolyte was replenished to the initial state using a fluid infusion pump at a constant rate.

**Computational Details**

All spin-polarized DFT calculations in this work were conducted using the Vienna *ab initio* simulation program (VASP)^[4]^. The electronic structures were calculated using the generalized gradient approximation (GGA) with the Perdew-Burke-Ernzerhof (PBE) functional^[5]^, while the ionic cores were described by the projector-augmented wave (PAW) potentials^[4b, 6]^. A plane-wave basis expansion cut-off energy of 400 eV was chosen. For model construction, a *p*(1 × 1) Ni_3_S_2_ (1 1 0) surface and a *p*(1 × 1) MoO_3_ (1 1 0) surface with three-layers were modeled. Moreover, a heterojunction structure where Mo_8_O_20_ was adsorbed on a three-layer Ni_3_S_2_ (1 1 0) surface were built. The top two layers were allowed to fully relax, while the bottom layer was held fixed to mimic the bulk region. A vacuum layer of ~20 Å was introduced to eliminate interaction between adjacent slabs. A 3 × 3 × 1 Monkhorst–Pack k-point mesh sampling was used for all optimizations. Equilibrium was achieved when forces on relaxed atoms and energies in self-consistent iterations became less than 0.05 eV Å^‒1^ and 10^‒5^ eV, respectively. The van der Waal (vdW) interaction was described by the DFT-D3 method^[7]^. Dipole corrections were applied to the surface normal direction.

The Gibbs free energy can be expressed as:

$$\Delta G=\Delta E+\Delta ZPE-T\cdot\Delta S$$

Where Δ*E* represents the reaction energy computed through the DFT methods. Δ*ZPE* and *T*Δ*S* correspond to the thermodynamic corrections of zero-point-energy (ZPE) and entropy (S) derived from vibrational partition function at 298.15 K.

**Proposed Mechanism**

The mechanism of the dynamic changes of MoS_2_ during HER was described in detail as follows:

Thermodynamically, the Pourbaix diagram^[8]^ reveals that the MoS_2_ exists as [MoO_4_^2−^ + S^2−^] under alkaline HER conditions, indicating the MoS_2_ is easily oxidized to MoO_4_^2−^. Besides, we can observe a broad oxidation peak under reducing potentials, also suggesting the electro-oxidation of the MoS_2_.

MoS_2_ + 8 OH^−^ → MoO_4_^2−^ + 2 S^2−^ + 4 H_2_O + 2 e^−^

Then, MoO_4_^2-^ was reduced and redeposited on the electrode surface as MoO_x_ under negative potential.

MoO_4_^2−^ + (4−x) H_2_O → MoO_x_ + (2x−6) e^−^ + (8−2x) OH^−^

According to the literature report^[9]^, the redeposited MoO_x_ establishes a dynamic equilibrium between dissolution and redeposition.


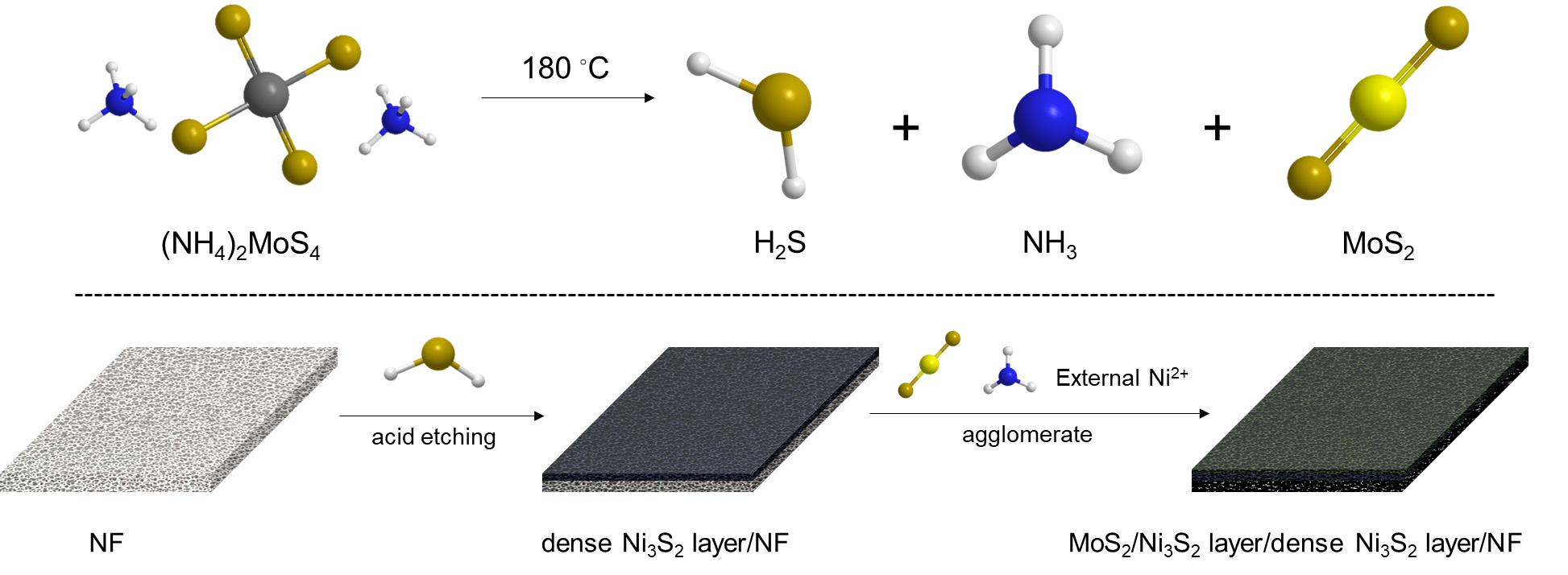


**Figure S1** Schematic illustration of the (NH_4_)_2_MoS_4_ decomposition and the proposed growth mechanism of the obtained electrode.


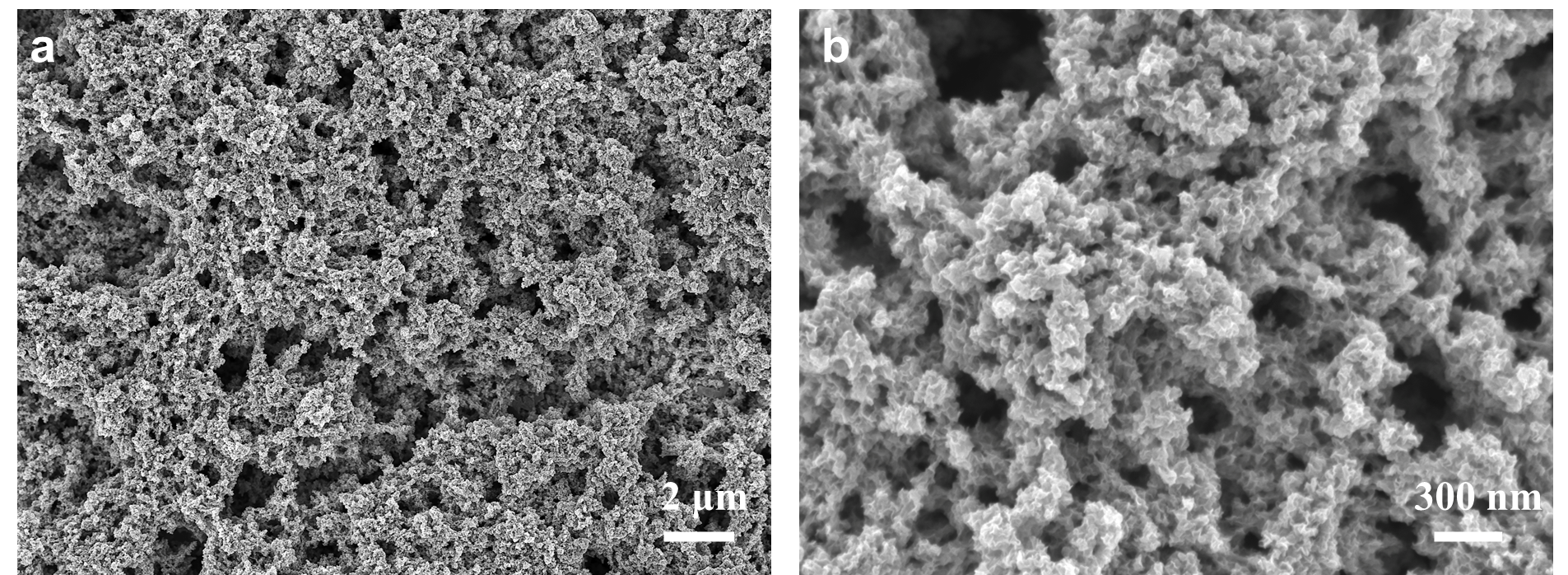


**Figure S2** SEM images of the as-prepared catalyst at different magnifications.


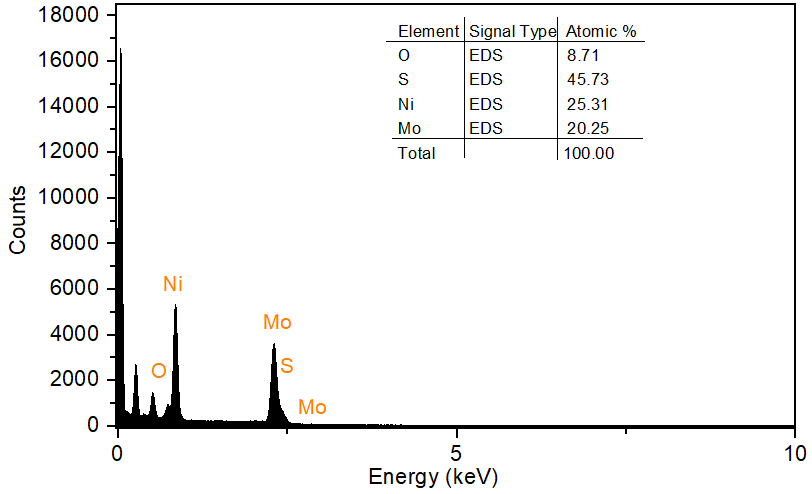


**Figure S3** SEM-EDS spectrum of the as-prepared MoS_2_/Ni_3_S_2_.

**Figure S4** SEM-EDS mapping of the selected region of the as-prepared MoS_2_/Ni_3_S_2_.


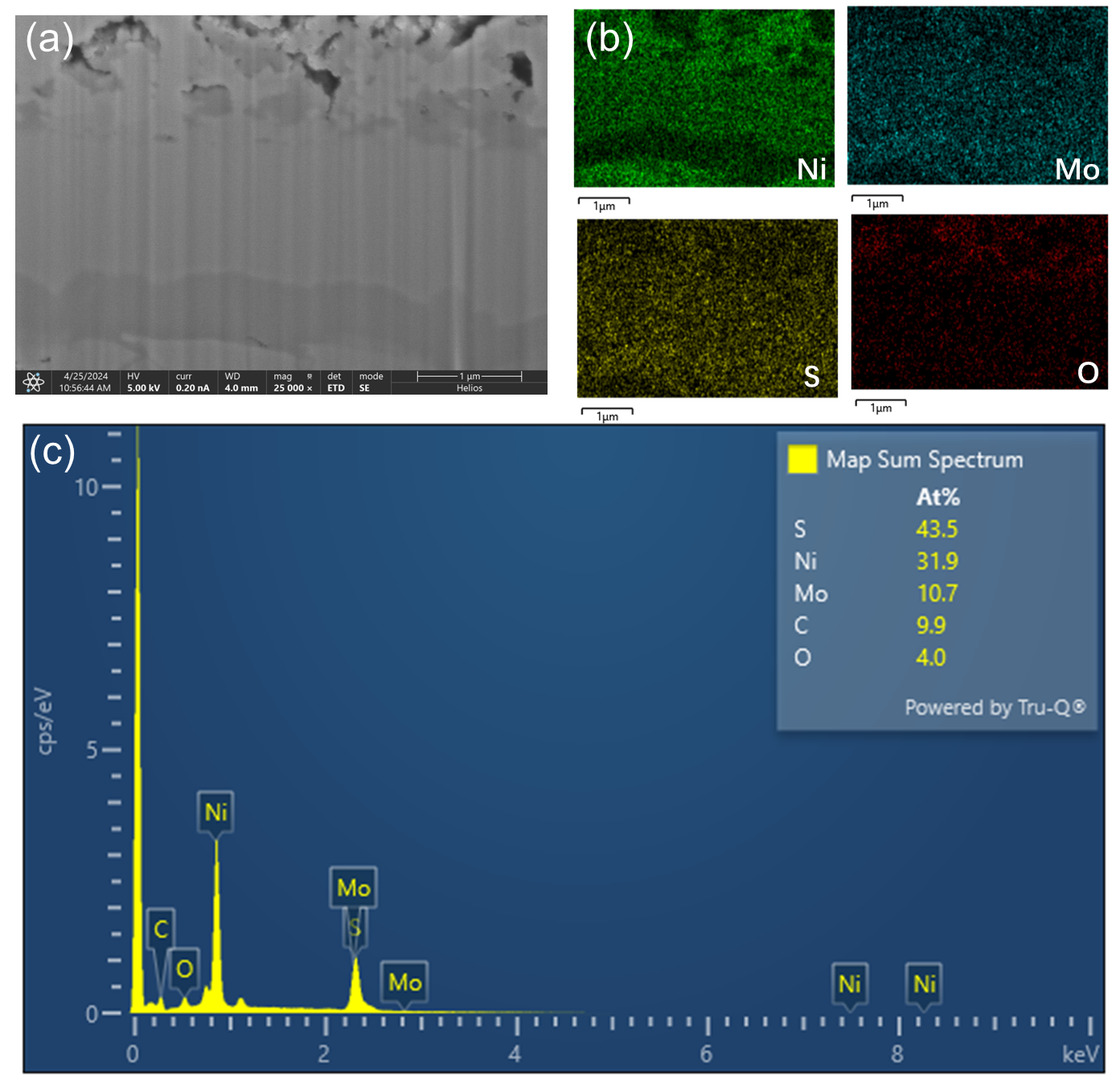


**Figure S5** (a) Linear scan SEM image; (b) SEM-EDS mapping of the selected region and (c) SEM-EDS spectrum of the as-prepared MoS_2_/Ni_3_S_2_.

**Figure S6** Linear scan SEM-EDS mapping of cross-section selected region of the as-prepared MoS_2_/Ni_3_S_2_.

**Figure S7** STEM-EDS mapping of the as-prepared MoS_2_/Ni_3_S_2_.

**Figure S8** Activation of the catalysts using 20 successive CV scans.

**Figure S9** SEM images of the activated MoO_x_/A-Ni_3_S_2_.


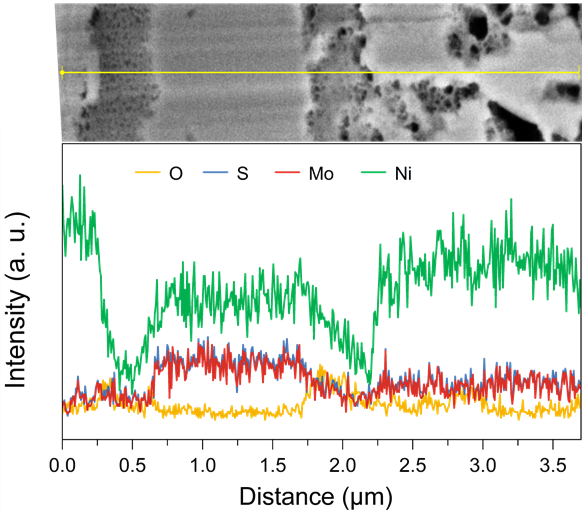


**Figure S10** Linear scan SEM-EDS mapping of cross-section selected region of the activated MoO_x_/A-Ni_3_S_2_.

**Figure S11** PXRD pattern of the MoO_x_/A-Ni_3_S_2_.

_
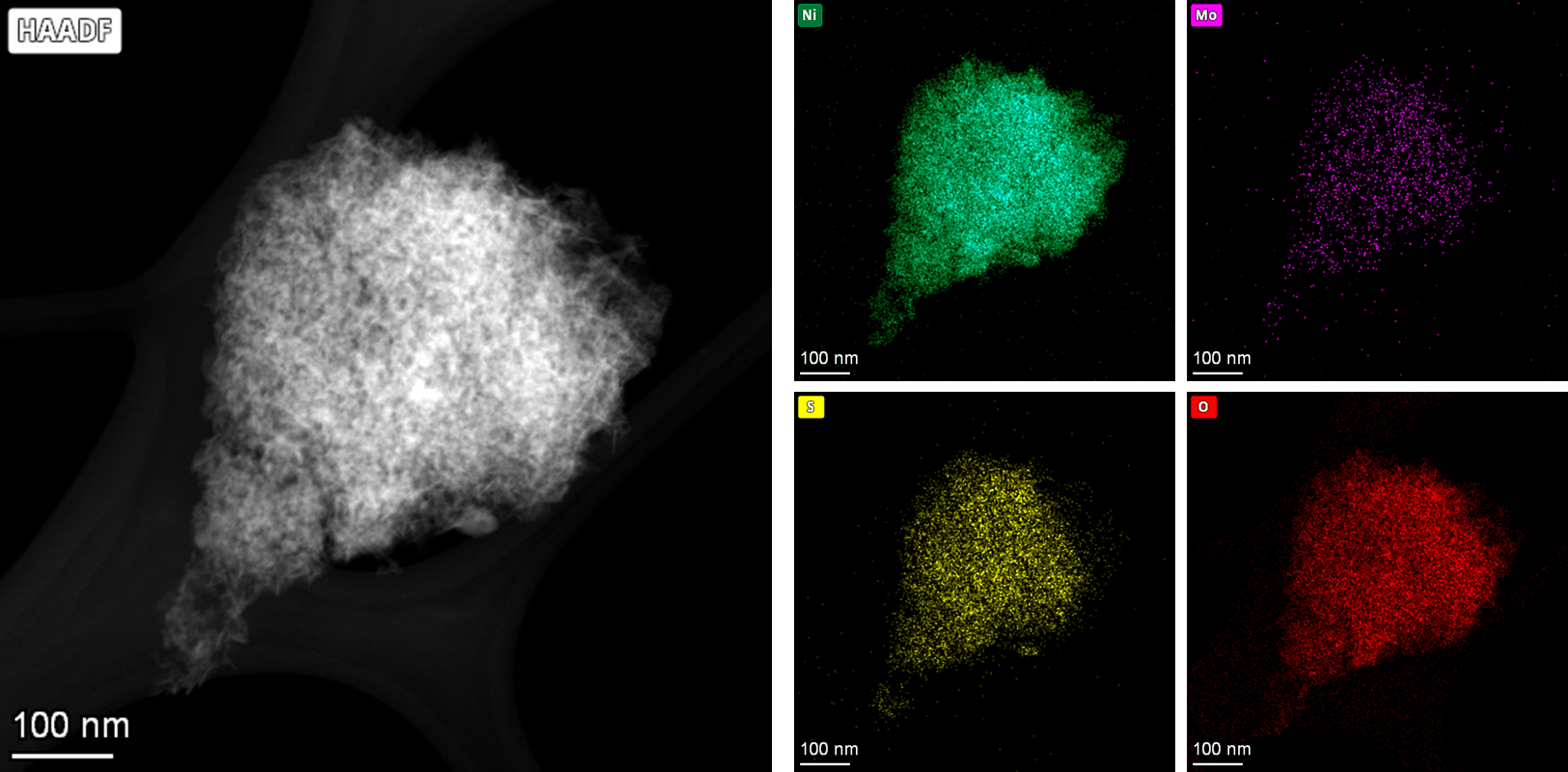
_

**Figure S12** HAADF-STEM and its corresponding element mapping of the MoO_x_/A-Ni_3_S_2_.

**Figure S13** TEM-EDS spectrum of the MoO_x_/A-Ni_3_S_2_.

**Figure S14** XPS survey of the as-prepared (a) MoS_2_/Ni_3_S_2_ and (b) MoO_x_/A-Ni_3_S_2_.


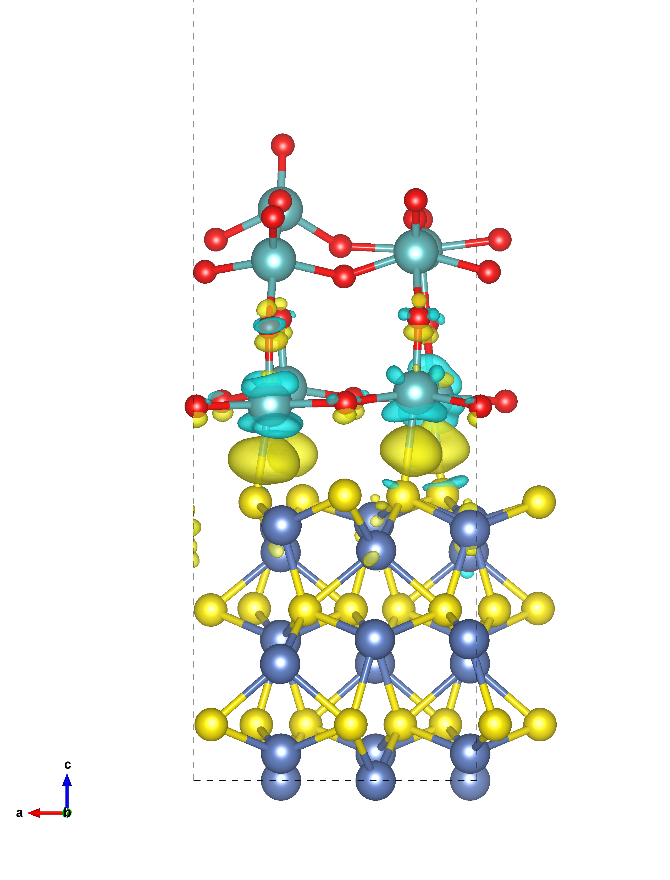


**Figure S15** Differential charge density of MoO_x_/A-Ni_3_S_2_. The iso-value is 0.005 e/Å^3^. Yellow represents the electron accumulation area, and blue represents the electron dissipation area. Blue, red, cyan, and yellow balls represent Ni, O, Mo, and S, respectively.


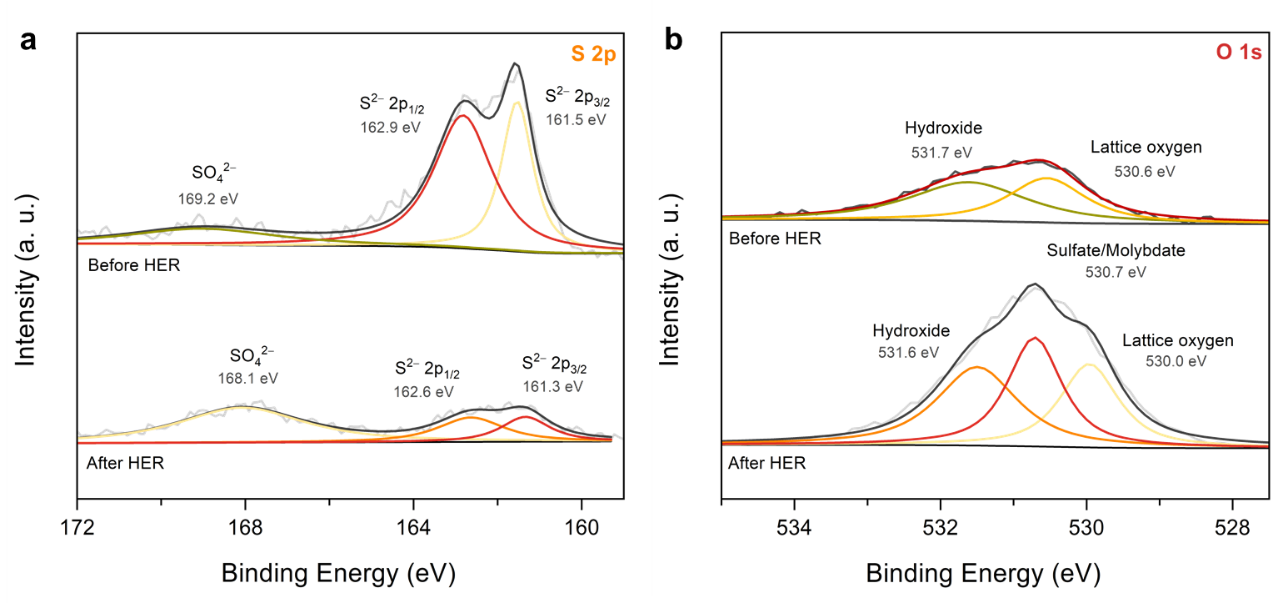


**Figure S16** Comparison of XPS fine spectra around (a) S 2p, (b) O 1s of the electrode before and after HER.

**Figure S17** EPR spectra of the MoO_x_/A-Ni_3_S_2_ and as-prepared MoS_2_/Ni_3_S_2_.


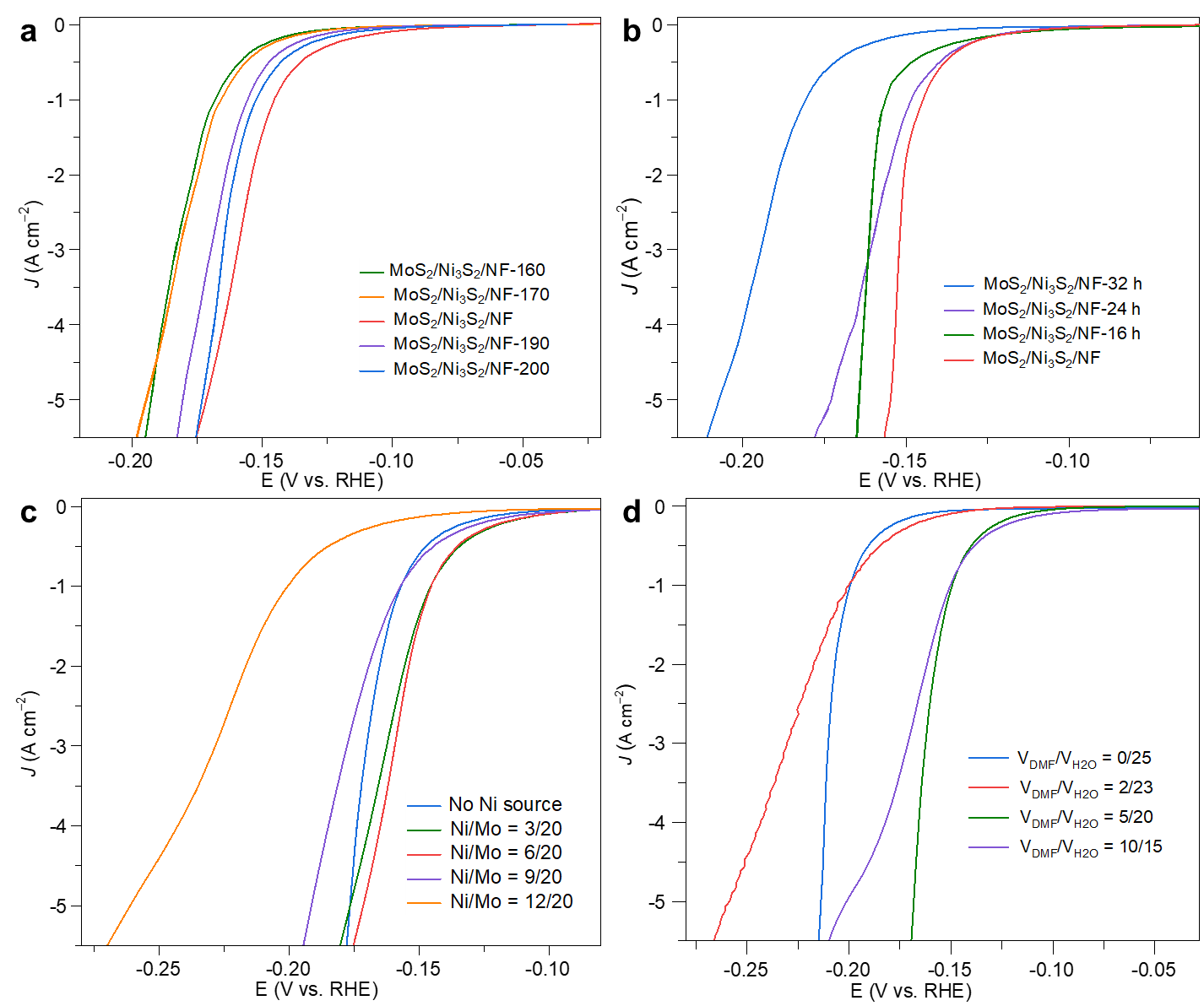


**Figure S18** Polarization curves of the samples obtained at (a) different hydrothermal temperatures, (b) different hydrothermal time (180 ^◦^C), (c) different Ni/Mo ratios, and (d) different amounts of DMF.

**Figure S19** Polarization curves (with 90% *iR*-compensation) of the MoO_x_/A-Ni_3_S_2_ and 40% Pt/C/NF in 1 M KOH.

**Figure S20** Faradic efficiency of H_2_ for MoO_x_/A-Ni_3_S_2_.


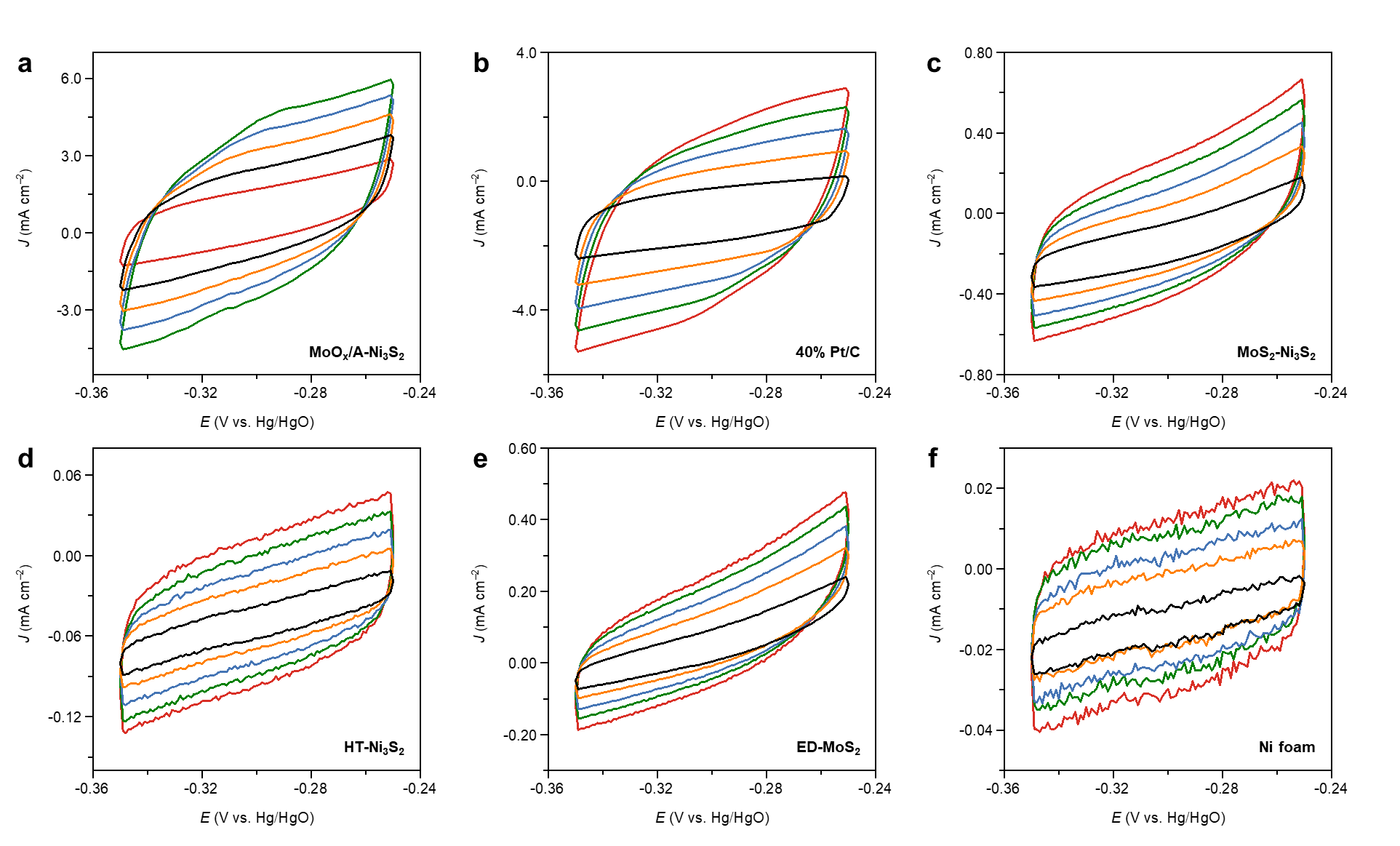


**Figure S21** CV curves at different scan rates (50, 100, 150, 200, 250 mV s^‒1^) of different electrodes.


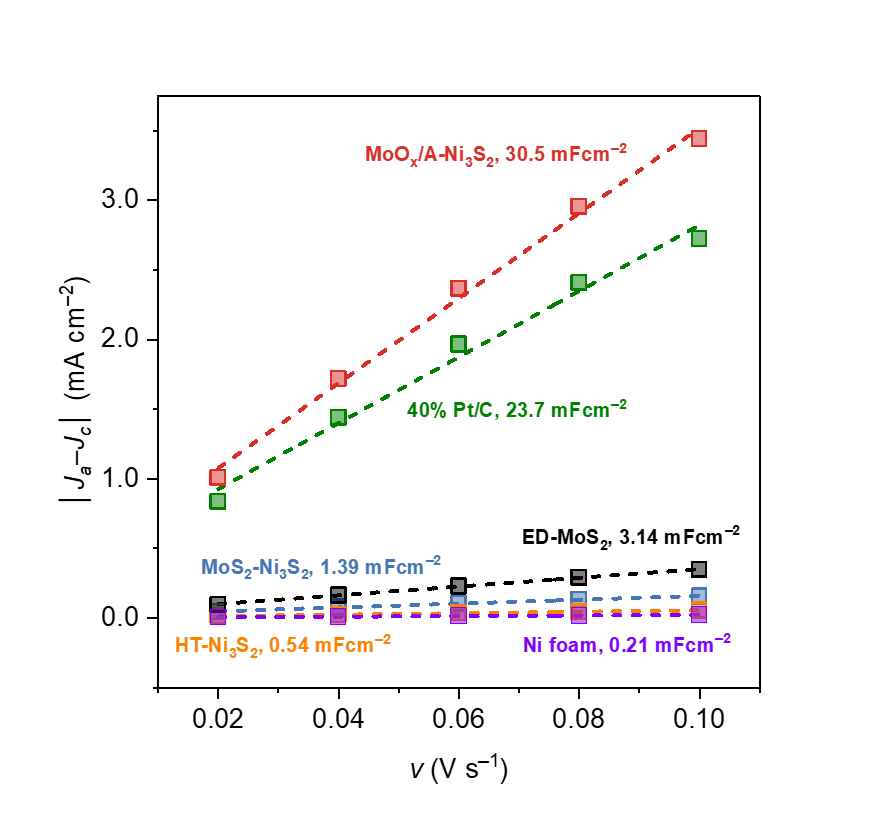


**Figure S22** The *C*_dl_ fitting plots of different electrodes.

**Figure S23** Nyquist plots of the different electrodes in 1 M KOH.

**
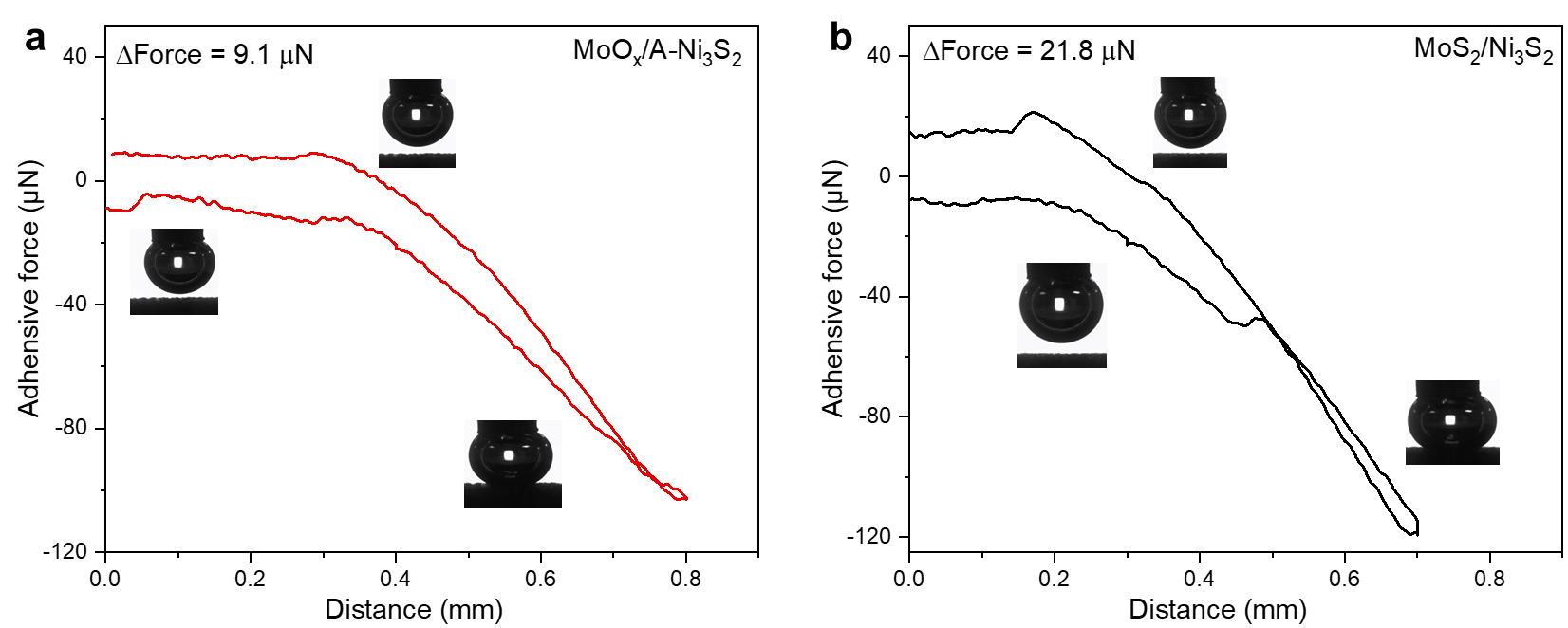
**

**Figure S24** Adhesive force of (a) MoO_x_/A-Ni_3_S_2_, (b) as-prepared MoS_2_/Ni_3_S_2_.


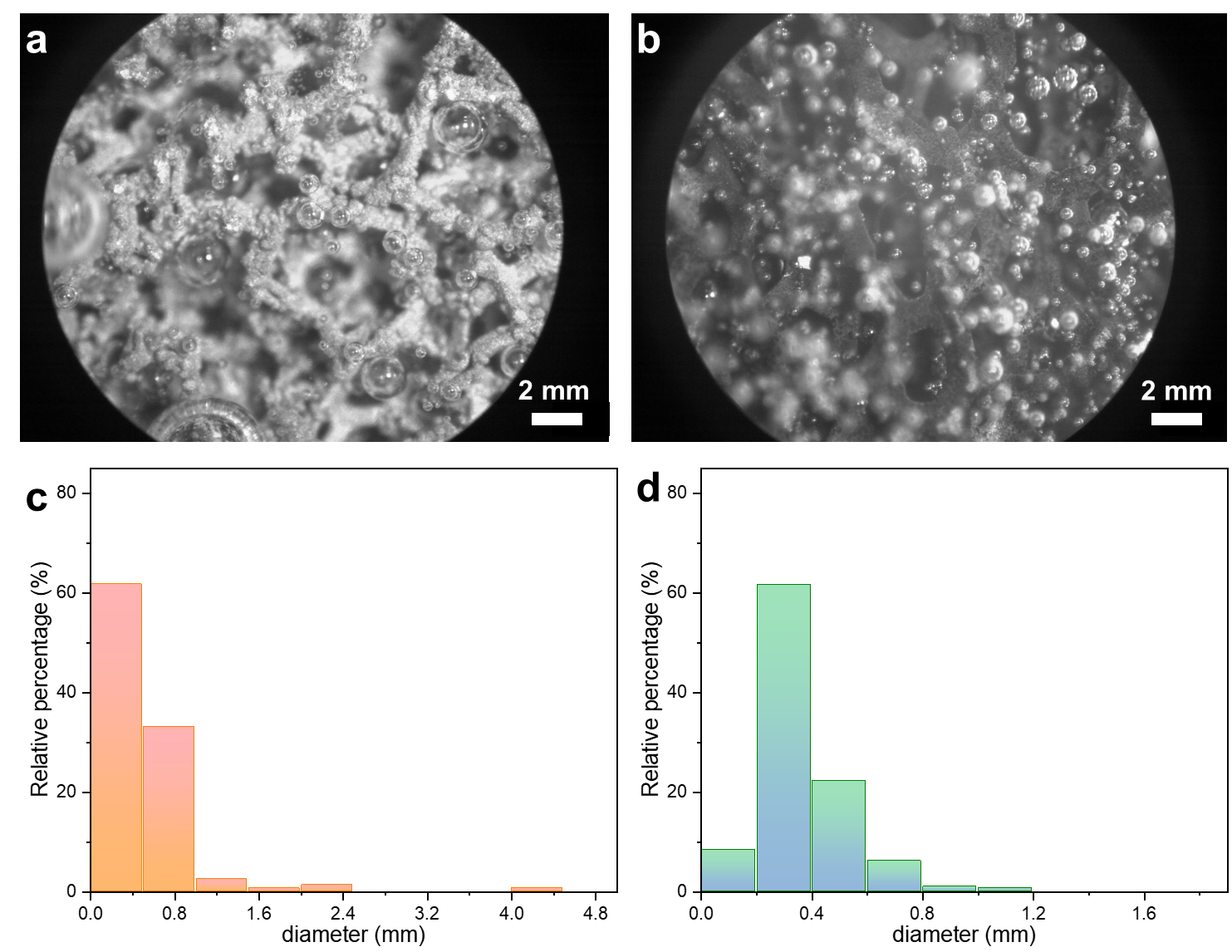


**Figure S25** Camera screenshots and Bubble detachment distribution from the as-prepared MoS_2_/Ni_3_S_2_ (a and c) and MoO_x_/A-Ni_3_S_2_ (b and d) at 50 mA cm^‒2^.


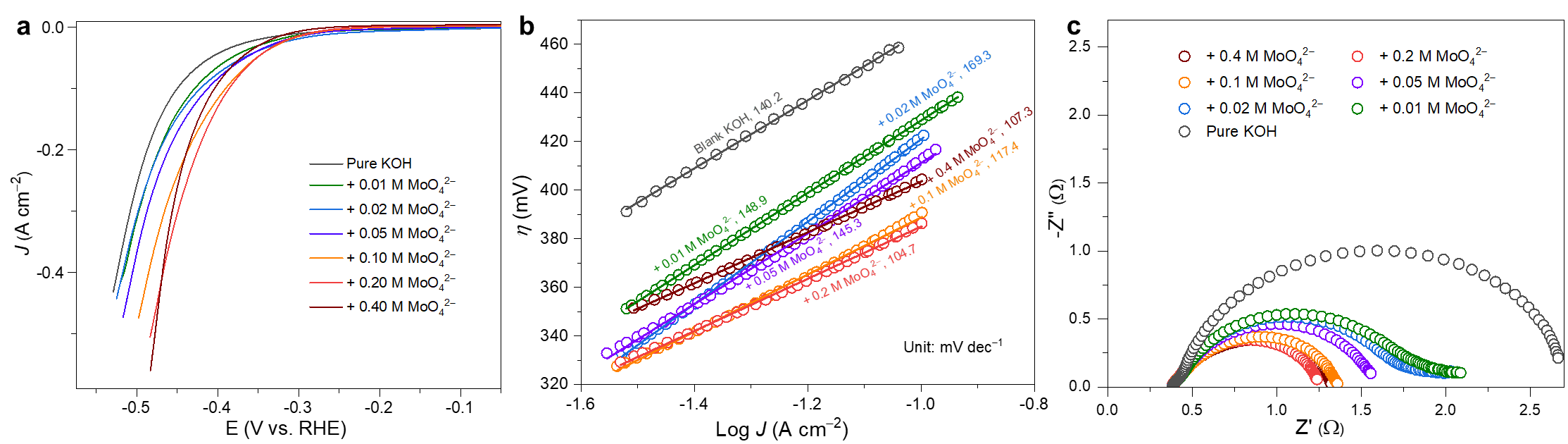


**Figure S26** (a) Polarization curves, (b) Tafel slopes, and (c) Nyquist plots of HT-Ni_3_S_2_ electrode in 1 M KOH with different concentrations of MoO_4_^2−^.

**Supplementary Notes:**

Extra MoO_4_^2−^ with different molar concentrations was added into the electrolyte when the HT-Ni_3_S_2_ electrode was applied as HER catalyst (**Figure S26**). As shown in **Figure S26a**, the extra addition of MoO_4_^2−^ can improve the HER activity of the Ni_3_S_2_ in 1 M KOH solution. It is obvious to observe that the HER activity of the Ni_3_S_2_ was gradually increased upon increasing the concentration of MoO_4_^2−^ from 0.01 M to 0.2 M and reached best activity with the MoO_4_^2−^ concentration of 0.2 M. Additionally, the Tafel slope (**Figure S26b**) declines from 140.2 to 104.7 mV dec^−1^ when increases the concentration of MoO_4_^2−^ from 0.01 M to 0.2 M, indicating the addition of Mo does improve the HER kinetics. Furthermore, Nyquist plots of the samples (**Figure S26c**) also illustrated that the arc diameter became smaller when the concentration of MoO_4_^2−^ increased from 0.01 M to 0.2 M, which proved that adding MoO_4_^2−^ can minimize the charge transfer of the Ni_3_S_2_.

**Figure S27** Chronopontimetric curve of the HT-Ni_3_S_2_ electrode at 1 A cm^−2^ in 1 M KOH solution and 1 M KOH + 0.2 M MoO_4_^2‒^.

**Supplementary Notes:**

As shown in **Figure S27**, the electrode showed inferior stability in 1 M KOH, deteriorating with a rate of 16.7 mV h^−1^ when electrolyzed at a current density of 1 A cm^−2^. After adding 0.2 M MoO_4_^2‒^, the electrode keeps steady during 35 h of electrolysis at a current density of 1 A cm^−2^.


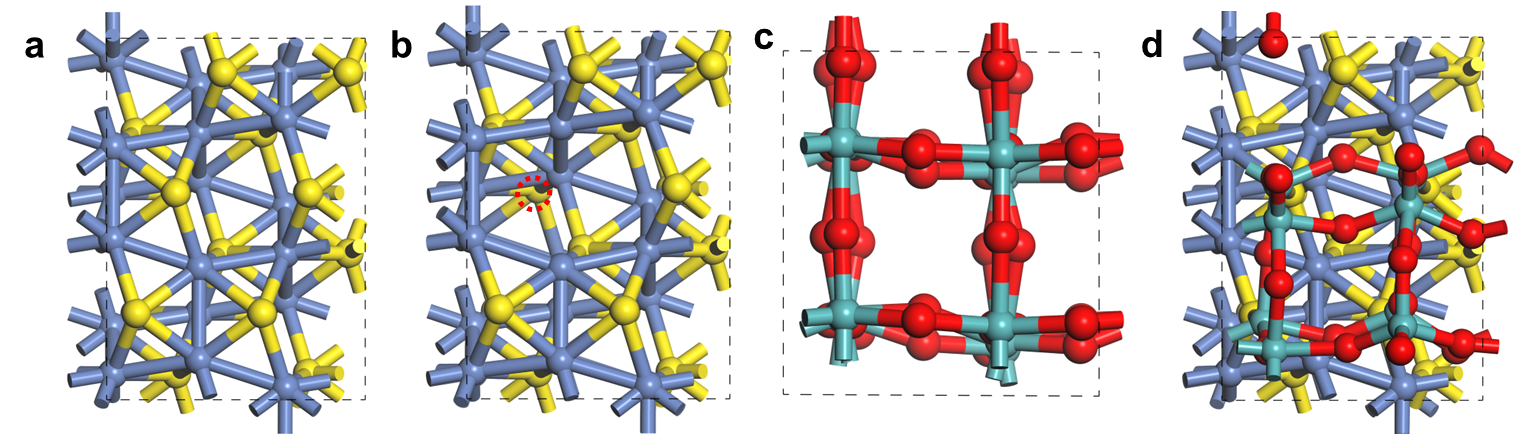


**Figure 28** Top view of (a) Ni_3_S_2_, (b) A-Ni_3_S_2_, (c) MoO_3_, (d) MoO_x_/ A-Ni_3_S_2_ slab models.

**Figure S29** Free energy diagrams for HER on (a) Ni_3_S_2_, (b) A-Ni_3_S_2_.


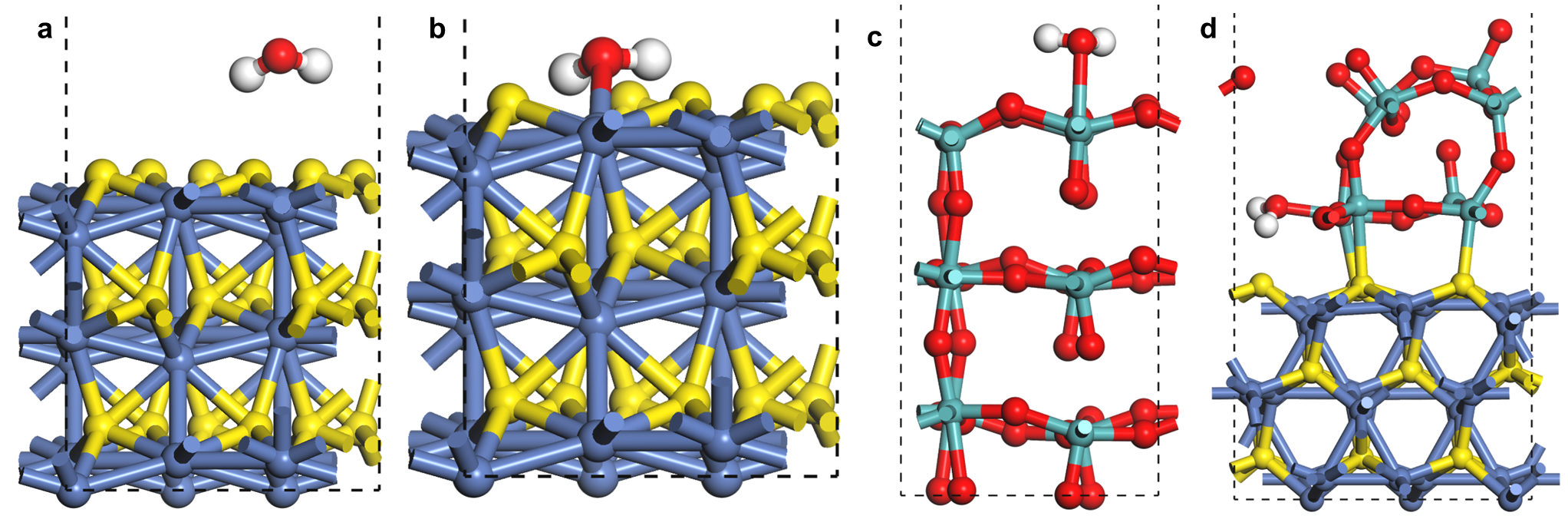


**Figure 30** Configurations of H_2_O adsorbed on (a) Ni_3_S_2_, (b) A-Ni_3_S_2_, (c) MoO_3_, (d) MoO_x_/ A-Ni_3_S_2_. White represents H.

**Figure 31** The adsorption free energies of H_2_O on (a) Ni_3_S_2_, (b) A-Ni_3_S_2_, (c) MoO_3_, (d) MoO_x_/ A-Ni_3_S_2_.

**Figure S32** The chronopontimetric curves of single-cell AEM-WE at a current density of 1 A cm^−2^ at 25 °C with flowing catholyte; T3, NiFe catalyst, and MoO_x_/A-Ni_3_S_2_ were used as AEM, anode and cathode, respectively.


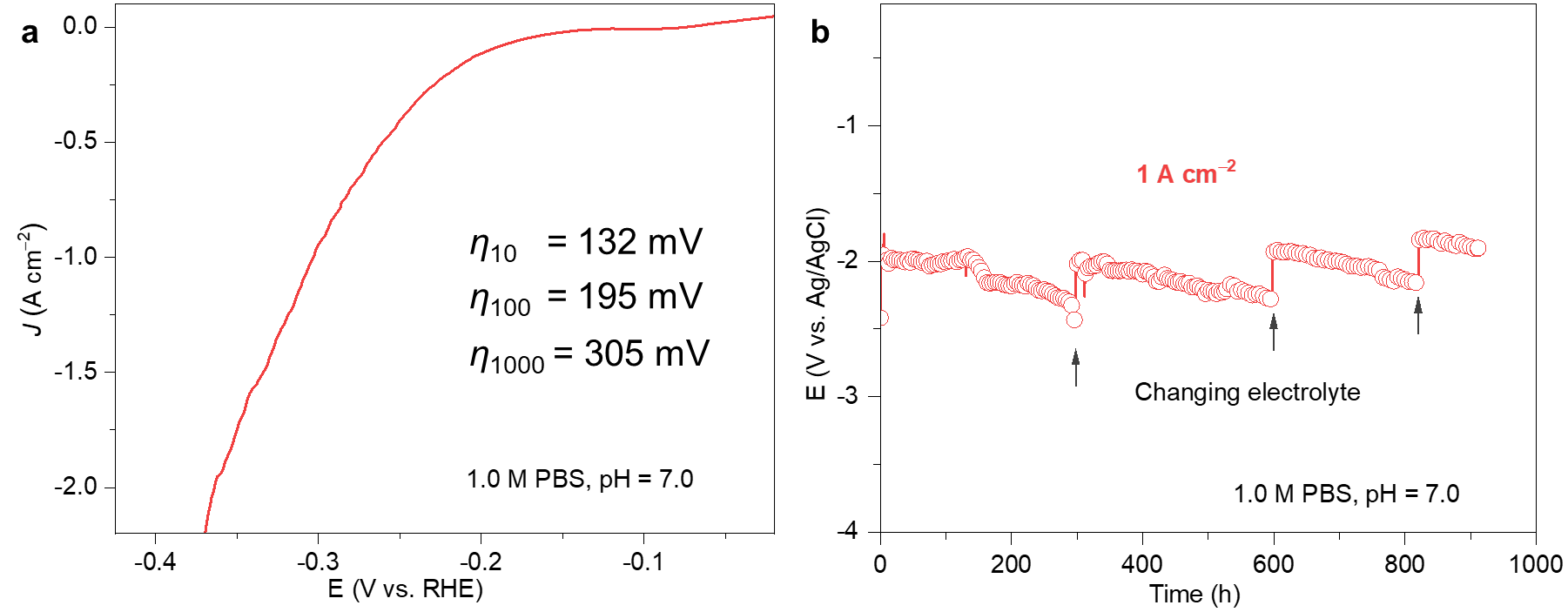


**Figure S33** (a) polarization curve and (b) chronopontimetric curve of the catalyst in 1.0 M PBS (pH = 7.0).


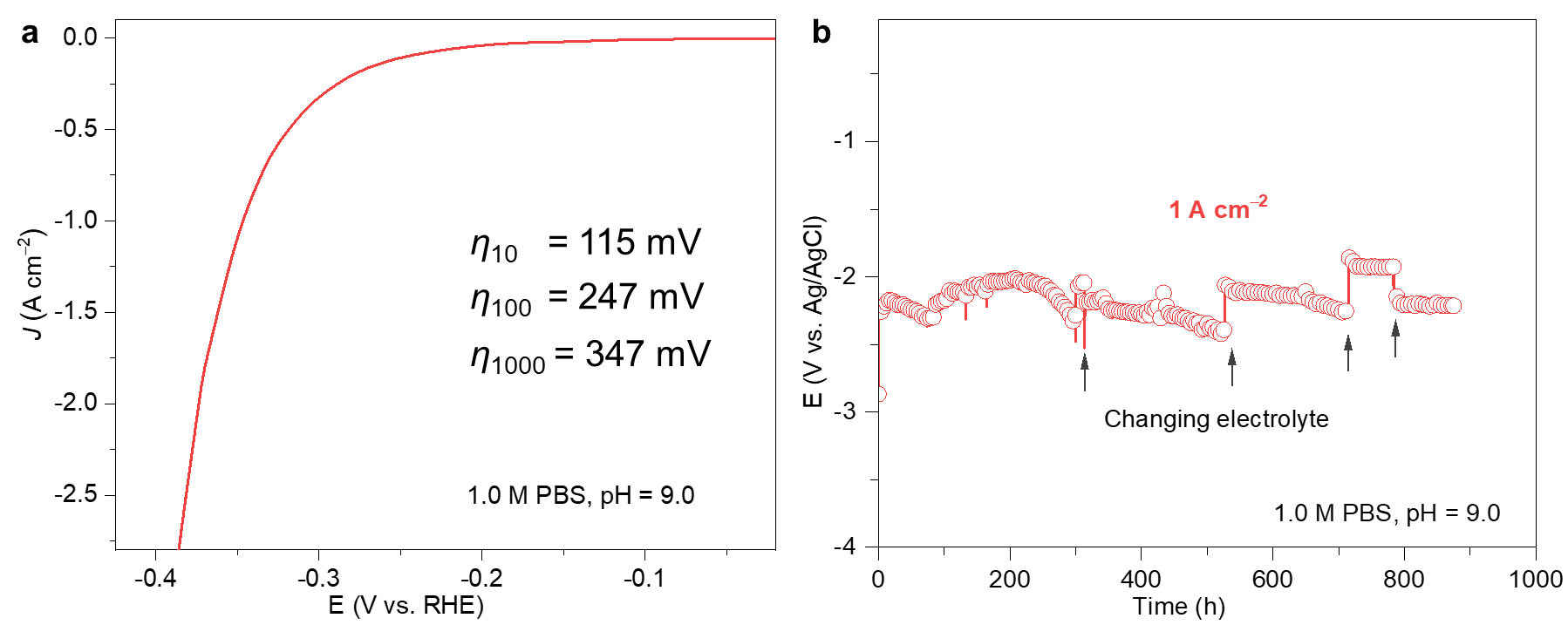


**Figure S34** (a) polarization curve and (b) chronopontimetric curve of the catalyst in 1.0 M PBS (pH = 9.0).


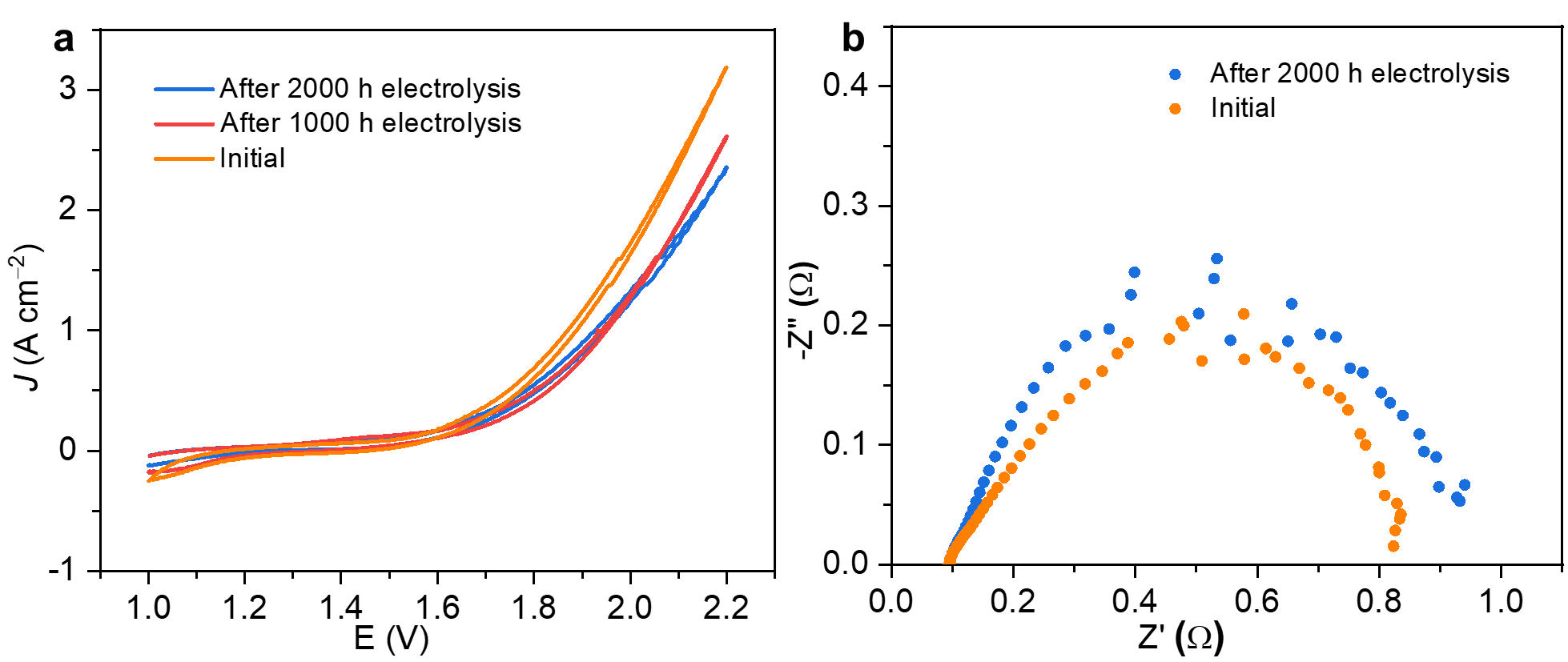


**Figure** **S35** (a) CV curves and (b) Nyquist plots of the AEM electrolyzer before and after 2000 h electrolysis.

**Figure S36** Time-dependent concentration of dissolved Mo, Ni, and Fe in the anolyte during AEM electrolysis at a current density of 1 A cm^−2^ at room temperature using dry cathode operation.

**Table S1** Brief conclusion of the HER performance of non-noble metal-based catalysts in 1 M KOH at high current density.

| **Catalyst** | ***j*/mA cm^‒2^** | ***η*/mV** | **Stability test** | **Ref.** |
| --- | --- | --- | --- | --- |
| MoO_x_/A-Ni_3_S_2_ | 100 | 105 | 1200 h @ 1000 mA cm^‒2^ | This work |
|  | 1000 | 145 |  |  |
| Ni_2(1−x)_Mo_2x_P | 100 | 150 | 25 h @ 10 mA cm^‒2^  25 h @ 100 mA cm^‒2^  20 h @ 500 mA cm^‒2^ | ^[10]^ |
|  | 1000 | 294 |  |  |
| Co-B-P/NF | 100 | 100 | 20 h @ 1000 mA cm^‒2^ | ^[11]^ |
|  | 1000 | 165 |  |  |
| Ni_2_P/NF | 100 | 249 | 24 h @ 240 to 650 mV of overpotential | ^[12]^ |
|  | 1000 | 306 |  |  |
| MoS_2_/Ni_3_S_2_ NW-NF | 100 | 150 | 12 h @ 200 mA cm^‒2^  12 h @ 500 mA cm^‒2^  12 h @ 1000 mA cm^‒2^ | ^[13]^ |
|  | 1000 | 200 |  |  |
| NiMoO_x_/NiMoS | 100 | 90 | 25 h @ 100 mA cm^‒2^  25 h @ 500 mA cm^‒2^ | ^[14]^ |
|  | 1000 | 236 |  |  |
| A-NiCo LDH/NF | 100 | 120 | 72 h @ 500 mA cm^‒2^  72 h @ 1000 mA cm^‒2^ | ^[15]^ |
|  | 1000 | 381 |  |  |
| Co- Mo_5_N_6_ | 100 | 100 | 10 h @ 50 mA cm^‒2^  10 h @ 1000 mA cm^‒2^  10 h @ 5000 mA cm^‒2^ | ^[16]^ |
|  | 1000 | 280 |  |  |
| h-NiMoFe | 100 | 43 | 10 h @ 600 mA cm^‒2^  10 h @ 1000 mA cm^‒2^  10 h @ 1500 mA cm^‒2^ | ^[17]^ |
|  | 1000 | 97 |  |  |
| MoNi_4_/MoO_3-x_ | 100 | 50 | 9 h @ 20 mA cm^‒2^  11 h @ 30 mA cm^‒2^ | ^[18]^ |
|  | 1000 | 153 |  |  |
| FeCoNiP_0_S_1_ | 100 | 135 | 40 h @ 10 mA cm^‒2^ | ^[19]^ |
|  | 1000 | 264 |  |  |
| N-NiMoS | 100 | 150 | 1000 h @ 20 mA cm^‒2^ | ^[20]^ |
|  | 1000 | 322 |  |  |
| MoNi_4_/SSW | 100 | 50 | 10 h @ 100 mA cm^‒2^ | ^[21]^ |
|  | 1000 | 161 |  |  |
| F-Co_2_P/Fe_2_P/IF | 100 | 152 | 10 h @ 500 mA cm^‒2^  10 h @ 1000 mA cm^‒2^  10 h @ 2000 mA cm^‒2^ | ^[22]^ |
|  | 1000 | 261 |  |  |
| F_0.25_C_1_CH/NF | 100 | 180 | 136 h @ 100 mA cm^‒2^ | ^[23]^ |
|  | 1000 | 256 |  |  |
| Ni@C-MoO_2_/NF | 100 | 150 | 196 h @ 1000 mA cm^‒2^ | ^[24]^ |
|  | 1000 | 250 |  |  |
| Ni_3_Sn_2_-NiSnO_x_ | 100 | 60 | 3000 h @ 1000 mA cm^‒2^  (in an AWE setup) | ^[25]^ |
|  | 1000 | 165 |  |  |
| Ni_3_S_2_@LiMoNiO_x_(OH)_y_ | 100 | ~130 | 100 h @ 1000 mA cm^‒2^ | ^[26]^ |
|  | 1000 | 365 |  |  |
| NM-IHJ-V | 100 | 250 | 200 h @ 2000 mA cm^‒2^ | ^[27]^ |
|  | 1000 | 360 |  |  |
| NiCoP@FeNi LDH | 100 | 138 | 30 h @ 100 mA cm^‒2^ | ^[28]^ |
|  | 1000 | 195 |  |  |
| B_4.7_-MoNi_4_/NF | 100 | 47.44 | 200 h @ 500 mA cm^‒2^ | ^[29]^ |
|  | 1000 | 135.2 |  |  |
| HC-MoS_2_/Mo_2_C | 100 | ~ 200 | 24 h @ 500 mA cm^‒2^ | ^[30]^ |
|  | 1000 | 412 |  |  |
| Ni_2_P-CoOOH | 100 | ~ 90 | 100 h @ 2000 mA cm^‒2^ | ^[31]^ |
|  | 1000 | 240 |  |  |
| (Ni−MoO_2_)@C/NF | 100 | 200 | Multi-current process:  10, 100, 1000, 1500 mA cm^‒2^  Sum: 300 h | ^[32]^ |
|  | 1000 | 260 |  |  |
| NiCoP/NF | 100 | 171 | 300 h @ 1000 mA cm^‒2^ | ^[33]^ |
|  | 1000 | 328 |  |  |
| NiCoP/MXene· | 100 | 164 | - | ^[34]^ |
|  | 1000 | 298 |  |  |

**Table S2 Fitting results of EIS data (Figure S24) of the catalysts**

|  | MoO_x_/A-Ni_3_S_2_ | 40% Pt/C/NF | HT-Ni_3_S_2_/NF | ED-MoS_2_/NF | MoS_2_-Ni_3_S_2_/NF | NF |
| --- | --- | --- | --- | --- | --- | --- |
| *R*_s_/Ω | 0.0493 | 0.0849 | 0.0918 | 0.128 | 0.0306 | 0.223 |
| *R*_ct_/Ω | 0.0781 | 11.26 | 0.283 | 2.64 | 0.0208 | 0.468 |
| CPE1-T | 1.072 | 0.0021 | 0.0080 | 0.0210 | 0.0531 | 0.00192 |
| CPE1-P | 0.925 | 0.953 | 0.912 | 0.996 | 0.0114 | 0.915 |
| R_mt_/Ω | 0.0911 | 134.2 | 16.49 | 3.596 | 0.125 | 33.89 |
| CPE2-T | 4.185 | 0.00105 | 0.0056 | 0.0631 | 0.452 | 0.00257 |
| CPE2-P | 0.921 | 0.958 | 0.918 | 0.937 | 0.926 | 0.915 |

**Table S3.** Brief conclusion of the single-cell AEMWE performance assembled by non-noble

metal catalysts using CCS methods in 1 M KOH.

| **Cathode** | | **Anode** | **Membrane** | | | | **T/**  **°C** | | ***j*/A cm****^‒2^** | | **E/V** | | | **Stability test** | | **Ref.** | | | |
| --- | --- | --- | --- | --- | --- | --- | --- | --- | --- | --- | --- | --- | --- | --- | --- | --- | --- | --- | --- |
| MoO_x_/A-Ni_3_S_2_ | | NiFe catalyst | T3 | | | | 80 | | 7.4 | | 2.0 | | | 2500 h @ 2.0 A cm^‒2^ (40 °C) | | This work | | | |
| MoNi/NF | | NiFe-LDH/NF | PVBC-MPy/35%  PEK-cardo | | | | 60 | | 0.50 | | 2.0 | | | 45 h^α^ @ 0.5 A cm^‒2^ | | ^[35]^ | | | |
| NiAlMo | | NiAlMo | HTM-PMBI | | | | 60 | | 1.0 | | 1.87^α^ | | | 12 h^α^ @ 1.0 A cm^‒2^ | | ^[36]^ | | | |
|  |  | NiAl |  |  |  |  |  |  | 1.0 | | 2.09^α^ | | | 154 h @ 1.0 A cm^‒2^ | |  |  |  |  |
| Ni–Fe–Co | | Ni–Fe–O*_x_* | PBI | | | | 60 | | 1.0 | | 1.90 | | | 100 h @ 1.0 A cm^‒2^ | | ^[37]^ | | | |
| NiFeCo | | NiFe_2_O_4_ | Sustainion® | | | | 60 | | 1.0 | | 2.01^α^ | | | **―** | | ^[38]^ | | | |
| Raney Ni | | NiFe_2_O_4_ | PTFE reinforced  Sustainion  Grade T | | | | 60 | | 1.0 | | 1.85 | | | 12180 h @  1.00 A cm^‒2^ | | ^[39]^ | | | |
| Ni-MoO_2_ | | Ni_0.6_Co_0.2_Fe_0.2_ | Fumapem-  3-PE-30 | | | | 50 | | 1.0 | | 1.97^α^ | | | 65 h @ 0.50 A cm^‒2^ in 0.1 M KOH | | ^[40]^ | | | |
| NiCoO-NiCo/C | | Cu_0.75_Co_2.25_O_4_ | Sustainion® X37–50 Grade T | | | | 50 | | 0.50 | | 1.85 | | | 10 h @ 0.44 A cm^‒2^ | | ^[41]^ | | | |
| Ni-Fe | | Ni-Fe | PFTP-13 | | | | 60 | | 1.0 | | 1.94 | | | 1000 h @ 0.5 A cm^‒2^ | | ^[42]^ | | | |
|  |  |  |  |  |  |  | 80 | |  |  | 1.83 | | | **―** | |  |  |  |  |
| MoNi_4_/MoO_2_/NF | | Ni_2_P@FePO_x_H_y_ | Sustainion® X37–50 | | | | 60 | | 1.0 | | 1.84 | | | 72 h @ 0.48 A cm^‒2^ | | ^[43]^ | | | |
| NiFeCo | NiFe_2_O_4_ | | | C-IL-100 | 80 | | | | 0.88 | | 2.2 | 8 h @ 0.10 A cm^‒2^ | | | | ^[44]^ | |  |  |
| M-Mo-CoP/CF | | NiFe-LDH/IF | FAA-3-50 | | | | 80 | | 1.0 | | 1.8 | | | 10 h@ 1.0 A cm^‒2^ | | ^[45]^ | | | |
| MoNi_4_/  MoO_2_/NF | | Ni_2_Fe_8_-Ni_3_S_2_/NF | | Sustainion® X37–50 Grade T | | | 60 | 1.0 | | | | 1.89 | | | 100 h @ 1.0 A cm^‒2^ | | | ^[46]^ |  |
|  |  |  |  |  |  |  | 80 |  |  |  |  | 1.65 | | | 18 h @ 1.0 A cm^‒2^ | | |  |  |
| NiMo/TP-4 | | NiFe/TP-4 | Sustainion® X37–50 | | | | 50 | | 10 | | 2.7 | | | 100 h @ 1.0 A cm^‒2^ | | ^[47]^ | | | |
| NiFeO*_x_* | | NiFe alloy | FAA-3-50 | | | | 70 | | 1 | | 1.8 | | | 500 h @ 0.5 A cm^‒2^ | | ^[48]^ | | | |

**Reference**

[1] Y. J. Noori, S. Thomas, S. Ramadan, D. E. Smith, V. K. Greenacre, N. Abdelazim, Y. Han, R. Beanland, A. L. Hector, N. Klein, G. Reid, P. N. Bartlett, C. H. Kees de Groot, *ACS Appl. Mater. Interfaces* **2020**, *12*, 49786-49794.

[2] S. Chen, Y. Li, B. Wu, Z. Wu, F. Li, J. Wu, P. Liu, H. Li, *Electrochim. Acta* **2018**, *275*, 40-49.

[3] G. L. Zhiheng Li, Linqin Wang, Husileng Lee, Jian Du, Tang Tang, Guoheng Ding, Rong Ren, Wenlong Li, Xing Cao, Shiwen Ding, Wentao Ye, Wenxing Yang, Licheng Sun, *Nat. Catal.* **2024**, *7*, 944-952.

[4] aG. Kresse, J. Furthmüller, *Phys. Rev. B* **1996**, *54*, 11169-11186; bG. Kresse, D. Joubert, *Phys. Rev. B* **1999**, *59*, 1758-1775.

[5] J. P. Perdew, K. Burke, M. Ernzerhof, *Phys. Rev. Lett.* **1996**, *77*, 3865-3868.

[6] P. E. Blöchl, *Phys. Rev. B* **1994**, *50*, 17953-17979.

[7] aS. Grimme, J. Antony, S. Ehrlich, H. Krieg, *J. Chem. Phys.* **2010**, *132*, 154104; bS. Grimme, S. Ehrlich, L. Goerigk, *J. Comput. Chem.* **2011**, *32*, 1456-1465.

[8] Z. Chen, C. Liu, L. Sun, T. Wang, *ACS Catal.* **2022**, *12*, 8936-8975.

[9] W. Du, Y. Shi, W. Zhou, Y. Yu, B. Zhang, *Angew. Chem., Int. Ed.* **2021**, *60*, 7051–7055.

[10] L. Yu, I. K. Mishra, Y. Xie, H. Zhou, J. Sun, J. Zhou, Y. Ni, D. Luo, F. Yu, Y. Yu, S. Chen, Z. Ren, *Nano Energy* **2018**, *53*, 492–500.

[11] H. Sun, X. Xu, Z. Yan, X. Chen, L. Jiao, F. Cheng, J. Chen, *J. Mater. Chem. A* **2018**, *6*, 22062–22069.

[12] X. Yu, Z.-Y. Yu, X.-L. Zhang, Y.-R. Zheng, Y. Duan, Q. Gao, R. Wu, B. Sun, M.-R. Gao, G. Wang, S.-H. Yu, *J. Am. Chem. Soc.* **2019**, *141*, 7537–7543.

[13] S. Xue, Z. Liu, C. Ma, H.-M. Cheng, W. Ren, *Sci. Bull.* **2020**, *65*, 123–130.

[14] P. Zhai, Y. Zhang, Y. Wu, J. Gao, B. Zhang, S. Cao, Y. Zhang, Z. Li, L. Sun, J. Hou, *Nat. Commun.* **2020**, *11*, 5462.

[15] H. Yang, Z. Chen, P. Guo, B. Fei, R. Wu, *Appl. Catal. B-Environ.* **2020**, *261*, 118240.

[16] F. Lin, Z. Dong, Y. Yao, L. Yang, F. Fang, L. Jiao, *Adv. Energy Mater.* **2020**, *10*, 2002176.

[17] Y. Luo, Z. Zhang, F. Yang, J. Li, Z. Liu, W. Ren, S. Zhang, B. Liu, *Energy Environ. Sci.* **2021**, *14*, 4610–4619.

[18] Y.-Y. Chen, Y. Zhang, X. Zhang, T. Tang, H. Luo, S. Niu, Z.-H. Dai, L.-J. Wan, J.-S. Hu, *Adv. Mater.* **2017**, *29*, 1703311.

[19] X. Wang, W. Ma, C. Ding, Z. Xu, H. Wang, X. Zong, C. Li, *ACS Catal.* **2018**, *8*, 9926–9935.

[20] C. Huang, L. Yu, W. Zhang, Q. Xiao, J. Zhou, Y. Zhang, P. An, J. Zhang, Y. Yu, *Appl. Catal. B-Environ.* **2020**, *276*, 119137.

[21] V. R. Jothi, K. Karuppasamy, T. Maiyalagan, H. Rajan, C.-Y. Jung, S. C. Yi, *Adv. Energy Mater.* **2020**, *10*, 1904020.

[22] X.-Y. Zhang, Y.-R. Zhu, Y. Chen, S.-Y. Dou, X.-Y. Chen, B. Dong, B.-Y. Guo, D.-P. Liu, C.-G. Liu, Y.-M. Chai, *Chem. Eng. J.* **2020**, *399*, 125831.

[23] L. Hui, Y. Xue, D. Jia, H. Yu, C. Zhang, Y. Li, *Adv. Energy Mater.* **2018**, *8*, 1800175.

[24] G. Qian, G. Yu, J. Lu, L. Luo, T. Wang, C. Zhang, R. Ku, S. Yin, W. Chen, S. Mu, *J. Mater. Chem. A* **2020**, *8*, 14545–14554.

[25] X. Wang, G. Long, B. Liu, Z. Li, W. Gao, P. Zhang, H. Zhang, X. Zhou, R. Duan, W. Hu, C. Li, *Angew. Chem., Int. Ed.* **2023**, *62*, e202301562.

[26] Q.-N. Ha, N. Susanto Gultom, M. Zefanya Silitonga, T. Negash Gemeda, D.-H. Kuo, *Chem. Eng. J.* **2023**, *467*, 143253.

[27] Y. Cheng, L. Zhang, S. Wang, M. Wang, C. Deng, Y. Sun, C. Yan, T. Qian, *ACS Nano* **2023**, *17*, 15504–15515.

[28] L. Yang, T. Yang, E. Wang, X. Yu, K. Wang, Z. Du, S. Cao, K.-C. Chou, X. Hou, *J. Mater. Sci. Technol* **2023**, *159*, 33–40.

[29] P. Liu, Y. Shi, X. Zhang, J. Yin, D. Zhang, T. Wang, J. Fei, T. Zhan, G. Li, J. Lai, L. Wang, *Appl. Catal. B-Environ.* **2024**, *341*, 123332.

[30] C. Zhang, Y. Luo, J. Tan, Q. Yu, F. Yang, Z. Zhang, L. Yang, H.-M. Cheng, B. Liu, *Nat. Commun.* **2020**, *11*, 3724.

[31] S. Zhang, W. Wang, F. Hu, Y. Mi, S. Wang, Y. Liu, X. Ai, J. Fang, H. Li, T. Zhai, *Nano-Micro Lett.* **2020**, *12*, 140.

[32] G. Qian, J. Chen, L. Luo, T. Yu, Y. Wang, W. Jiang, Q. Xu, S. Feng, S. Yin, *ACS Sustainable Chem. Eng.* **2020**, *8*, 12063–12071.

[33] L. He, Z. Cai, D. Zheng, L. Ouyang, X. He, J. Chen, Y. Li, X. Guo, Q. Liu, L. Li, W. Chu, S. Zhu, X. Sun, B. Tang, *J. Mater. Chem. A* **2024**, *12*, 2680–2684.

[34] H.-J. Niu, C. Huang, T. Sun, Z. Fang, X. Ke, R. Zhang, N. Ran, J. Wu, J. Liu, W. Zhou, *Angew. Chem., Int. Ed.* **2024**, *63*, e202401819.

[35] H. Li, M. R. Kraglund, A. K. Reumert, X. Ren, D. Aili, J. Yang, *J. Mater. Chem. A* **2019**, *7*, 17914–17922.

[36] L. Wang, T. Weissbach, R. Reissner, A. Ansar, A. S. Gago, S. Holdcroft, K. A. Friedrich, *ACS Appl. Energy Mater.* **2019**, *2*, 7903–7912.

[37] I. Vincent, E.-C. Lee, H.-M. Kim, *RSC Adv.* **2020**, *10*, 37429–37438.

[38] I. V. Pushkareva, A. S. Pushkarev, S. A. Grigoriev, P. Modisha, D. G. Bessarabov, *Int. J. Hydrogen Energy* **2020**, *45*, 26070–26079.

[39] B. Motealleh, Z. Liu, R. I. Masel, J. P. Sculley, Z. Richard Ni, L. Meroueh, *Int. J. Hydrogen Energy* **2021**, *46*, 3379–3386.

[40] A. Y. Faid, A. O. Barnett, F. Seland, S. Sunde, *ACS Appl. Energy Mater.* **2021**, *4*, 3327–3340.

[41] Y. S. Park, J. Jeong, Y. Noh, M. J. Jang, J. Lee, K. H. Lee, D. C. Lim, M. H. Seo, W. B. Kim, J. Yang, S. M. Choi, *Appl. Catal. B-Environ.* **2021**, *292*, 120170.

[42] N. Chen, S. Y. Paek, J. Y. Lee, J. H. Park, S. Y. Lee, Y. M. Lee, *Energy Environ. Sci.* **2021**, *14*, 6338–6348.

[43] A. Meena, P. Thangavel, D. S. Jeong, A. N. Singh, A. Jana, H. Im, D. A. Nguyen, K. S. Kim, *Appl. Catal. B-Environ.* **2022**, *306*, 121127.

[44] X. Wang, R. G. H. Lammertink, *J. Mater. Chem. A* **2022**, *10*, 8401–8412.

[45] H. Li, R.-Y. Fan, X.-Y. Zhang, M.-D. Tu, J.-F. Huang, Y.-M. Chai, B. Dong, *Electrochim. Acta* **2023**, *472*, 143429.

[46] G. Ding, H. Lee, Z. Li, J. Du, L. Wang, D. Chen, L. Sun, *Adv. Energy Sustainability Res.* **2023**, *4*, 2200130.

[47] J. Hyun Oh, G. Ho Han, J. Kim, J. Eun Lee, H. Kim, S. Kyung Kang, H. Kim, S. Wooh, P. Soo Lee, H. Won Jang, S. Young Kim, S. Hyun Ahn, *Chem. Eng. J.* **2023**, *460*, 141727.

[48] S. Park, J. E. Park, G. Na, C. Choi, Y.-H. Cho, Y.-E. Sung, *ACS Appl. Energy Mater.* **2023**, *6*, 8738–8748.
